# Simulating exposure-related behaviors using agent-based models embedded with needs-based artificial intelligence

**DOI:** 10.1038/s41370-018-0052-y

**Published:** 2018-09-21

**Authors:** Namdi Brandon, Kathie L. Dionisio, Kristin Isaacs, Rogelio Tornero-Velez, Dustin Kapraun, R. Woodrow Setzer, Paul S. Price

**Affiliations:** 1 National Exposure Research Laboratory, Office of Research and Development, United States Environmental Protection Agency, RTP, NC USA; 2 National Center for Environmental Assessment, Office of Research and Development, United States Environmental Protection Agency, RTP, NC USA; 3 National Center for Computational Toxicology, Office of Research and Development, United States Environmental Protection Agency, RTP, NC USA

**Keywords:** Agent-based model, Exposure-related behavior, Simulation, Artificial-intelligence

## Abstract

Exposure to a chemical is a critical consideration in the assessment of risk, as it adds real-world context to toxicological information. Descriptions of where and how individuals spend their time are important for characterizing exposures to chemicals in consumer products and in indoor environments. Herein we create an agent-based model (ABM) that simulates longitudinal patterns in human behavior. By basing the ABM upon an artificial intelligence (AI) system, we create agents that mimic human decisions on performing behaviors relevant for determining exposures to chemicals and other stressors. We implement the ABM in a computer program called the Agent-Based Model of Human Activity Patterns (ABMHAP) that predicts the longitudinal patterns for sleeping, eating, commuting, and working. We then show that ABMHAP is capable of simulating behavior over extended periods of time. We propose that this framework, and models based on it, can generate longitudinal human behavior data for use in exposure assessments.

## Introduction

Traditionally, exposure assessors and modelers obtain information about exposure-related behaviors by surveying individuals about their daily activities [1]. Surveyed individuals complete diaries that capture a range of activities over time [2–4]; the United States Environmental Protection Agency (U.S. EPA) has developed a database of such information for use in exposure assessment, called the Consolidated Human Activity Database (CHAD) [5]. However, collecting representative amounts of survey data is difficult and labor-intensive, especially for durations longer than 1 day. In addition, surveys cannot collect information of which the individual is either unaware (e.g., use of products by another nearby individual or the behavior of children) or is too time-consuming to report (e.g., amounts of each consumer product used in a given day). To avoid these difficulties, we have developed a method that models exposure-related behavior through use of agent-based models.

Agent-based models (ABMs) simulate the actions and interactions of autonomous agents within a system. Within the context of ABMs, the term “agent” represents a simulated being with agency and thus differs from the traditional definition of the term used in chemical risk assessments. The goal of an ABM is to gain explanatory insights into the behavior of complex processes by simulating the behavior of agents obeying a set of rules. Over time, the interaction between rules and agents can often lead to emergent behavior useful in modeling systems. ABMs combine elements of decision theory, computational sociology, and Monte Carlo methods, and are widely used in a range of research areas including sociology, biology, and ecology [6–12]. Here, we present a framework that uses methods from both the ABM and the artificial intelligence (AI) communities in order to model human behavior for simulating daily activities.

Needs-based AI is a method of decision-making used in ABMs based on agents’ satisfying various competing needs [13]. In needs-based AI, the agent considers multiple motivations (e.g. the need to eat, sleep, commute, work, etc.) linked to corresponding actions. The decisions of an agent in choosing behaviors based on competing, time-varying needs generate the pattern of actions. By modeling how human needs change over time and how humans react to those needs, we are able to simulate human behavior. This approach allows the individual to have a large number of competing needs, and therefore can explain a wide range of behaviors.

The decision to use a needs-based AI framework within the proposed ABM is based on the concept that humans make their decisions to take actions in order to fulfill needs. That is, when a need is in an “unsatisfied” state, a person will attempt to perform an action that sends the respective need to a “satisfied” state. For example, if a person is hungry (a state where the need to eat is unsatisfied), a person will seek an action, eating, which in turn will bring the need to a satisfied state. The concept that needs govern human decision-making was first proposed by Maslow [14]. Maslow attempted to explain human behavior by organizing needs into a hierarchy from basic physiological needs that address survival to increasingly complex needs that address personal fulfillment. The proposed ABM addresses the basic physiological and societal needs that are a high priority for individuals. More specifically, we consider four behaviors in adults: sleeping, eating, working, and commuting. These behaviors are chosen because people spend large portions of their time engaged in them [15] and they are relevant for determining other exposure-related behaviors.

In what follows, we present the ABM components and the underlying algorithms. Afterwards, we describe a computational model of longitudinal variation in activity. We then apply the model to a case study that simulates week-long daily activity of a working adult. Lastly, we give a summary of our findings and provide a brief discussion concerning our research.

## Methods

We now present the methods behind the ABM. We start by giving an overview of the model components. Next, we present how to model the decision-making process for choosing behaviors. Afterwards, we present the creation of a model of variation in longitudinal activity patterns followed by a description of parameterizing the longitudinal behavior.

### Model components

The following is an overview of the various components used to implement a needs-based AI system. The reader is referred to Zubek [13] for a further explanation of the concepts presented.

### Agent

An agent is an individual with control over its actions.

### Need

A need is a time-varying characteristic of an agent and is the basis of the motivation of the agent to perform an action. Examples of needs include the desires to rest, to eat, to earn an income, and to change location. These needs are modeled in the example ABM and are referred to in the rest of this paper as *Rest*, *Hunger*, *Income*, and *Travel*, respectively. We assume that, without interventions (actions) on the part of the agent, the magnitudes of all needs change over time from a satisfactory state towards an unsatisfactory state. Reaching such a state triggers an agent to select an action that restores the respective need to a satisfactory state.

### Activity

Activities are actions that an agent adopts in order to address unsatisfied needs. Activities have three properties. First, activities occur in specific environments (see Table 1). Second, activities begin at a specific point in time and have a finite duration. Third, an activity may impact multiple needs. In this version of the model, an agent can perform only one activity at a time. Unless otherwise stated, an activity inhibits decision-making during its duration.

**Table 1:** Components of the example ABM

Need	Activity	Object	Environment	Mathematical model
Rest	Sleep	Bed	Residence	Linear function
Hunger	Eat breakfast	Food	Residence	Linear function
Hunger	Eat lunch	Food	Residence and workplace	Linear function
Hunger	Eat dinner	Food	Residence	Linear function
Income	Work	Occupational objects	Workplace	Step function
Travel	Commute to work	Transport	Outdoors	Step function
Travel	Commute from work	Transport	Outdoors	Step function

### Linked activity

Some activities are inherently coupled to other activities (e.g., commuting to or from work is coupled to the start or end of working, respectively). We denote these activities as *linked activities*.

### Object and environment

Objects are items that an agent uses in order to perform an activity to address a need; the objects may be consumer products. Objects communicate with agents by advertising their value to individual agents. Environments are discrete collections of objects that can include one or more agents. An agent may move between environments, but can only be in one environment at any given moment. An agent may only interact with objects that share the agent’s current environment.

The components of the model are summarized in Table 1.

### Satiation

There are two quantities defining each need: its satiation *n* expressed as a function of time *n(t)* and its threshold parameter *λ*. The *satiation* is a measure of how satisfied an individual is with respect to a specific need. Satiation has a range [0,1] (*n(t)* ∈ [0,1]) where a value of 0 indicates the most unsatisfied state and 1 the most satisfied state. Satiation decreases over time unless an agent performs an activity that causes the satiation to increase.

The threshold *λ* controls how unsatisfied an agent must be in order for the agent to feel the desire to address the corresponding need. Alternatively said, the parameter *λ* acts as a trigger to initiate an activity [16]. As long as the satiation exceeds *λ* (*n*(*t*) > *λ*), an agent has no desire to address a need; the agent seeks activities only when (*n*(*t*) ≤ *λ*). This reflects the fact that people normally address needs only when they cross some threshold of satiation.

In this ABM, satiation declines over time in two ways. The first type of decline depends on the amount of time since the need was last satisfied. Examples of such needs are *Rest* and *Hunger*. We can model people as fully energized when waking and becoming increasingly tired throughout the day. In this model the decay rate is assumed to be a constant. Mathematically, it’s represented as

1 $$m_{{\mathrm{{decay}}}} = - \frac{{1 - \lambda }}{{{\mathrm{\Delta }}t_{{\mathrm{{decay}}}}}},$$

where *m*_decay_ is the decay rate and Δ*t*_decay_ is the minimum amount of time for the need to go from a fully satisfied state to an unsatisfied state. In this case, the decaying behavior of the need is modeled as a linear function. However, the decaying behavior may be modeled as any monotonically decreasing function.

The second type of decline resembles step functions. Needs described by these satiation functions represent scheduled behaviors, where the agent has committed to perform an action (that will satisfy a long-term need) at a specific time (e.g., working for an organization or attending a school). Thus these functions depend on the current time of day, rather than the amount of time from a prior event. The satiation *n*(*t*) for these needs may be mathematically modeled as

2 $$n\left( t \right) = \left\{ {\begin{array}{*{20}{c}} {1,t \ < \ t_0} \\ {\eta ,t \ge t_0} \end{array}} \right.$$

where *t* is the current time and *t*_0_ corresponds to the time of day that the need becomes unsatisfied. The parameter *η* has value 0 ≤ *η* ≤ *λ* and is the value of satiation once the corresponding motivation is triggered.

The satiation may increase due to an action in two ways. For a linearly behaving need, the recovery rate of the satiation of a need associated with an action is a constant defined as

3 $$m_{{\mathrm{{recover}}}} = \frac{{1 - \lambda }}{{{\mathrm{\Delta }}t_{{\mathrm{{recover}}}}}},$$

where *m*_recover_ is the recovery rate and Δ*t*_recover_ is the minimum amount of time required to go from the unsatisfied state equal to *λ* to a completely satisfied state (*n* = 1) as the result of performing an activity. We can mathematically express the required duration to achieve full recovery through such an action as

4 $${\mathrm{\Delta }}t_{{\mathrm{{activity}}}} = \frac{{1 - n\left( t \right)}}{{m_{{\mathrm{{recover}}}}}}.$$

Second, if the need resembles a step function, an action may increase the satiation as

5 $$n\left( t \right) = \left\{ {\begin{array}{*{20}{c}} {\eta ,t \hskip1.5pt < \hskip1.5pt t_0} \\ {1,t \ge t_0} \end{array}} \right.$$

where *t*_0_ is the time at which the need is satisfied.

Table 1 summarizes the mathematical model of each need modeled in the ABM.

### Score

Scores are a function of need-specific weights that measure the amount of urgency a need has. The weight function *W(n)* has the property that it gives low values of satiation *n* higher values of importance. That is, *W(n)* is inversely proportional to *n*. This relationship captures the concept that lower values of *n(t)* should correspond to more urgent desires. The weight function in this model is defined as

6 $$W(n) = \left\{ {\begin{array}{*{20}{c}} {0,n \hskip1.5pt > \hskip1.5pt \lambda } \\ {\frac{1}{{n\; +\; {\it{\epsilon }}}},\,n \le \lambda } \end{array}} \right.$$

where *∈* > 0 is a constant that prevents division by zero.

An agent chooses a particular activity by reviewing all activities advertised by the available objects and then by comparing the respective scores of all advertisements. First, note that performing an advertised activity should change an affected need *n(t)* by some advertised amount *δ*. When the advertised activity is completed in the future, the value of satiation will change. That is,

$$n(t + {\mathrm{\Delta }}t) = n(t) + \delta .$$

Second, we base the advertised score not only on the agent’s current state of need *n(t)* but also on the agent’s future state n(*t + Δt*), under the presumption that the respective activity of duration Δ*t* is chosen. Given the current state of a need, the advertised score *S*(*n*) from an activity is

7 $$S\left( n \right) = \left\{ {\begin{array}{*{20}{c}} {0,n > \lambda } \\ {W\left( {n\left( t \right)} \right) - W\left( {n\left( {t + {\mathrm{\Delta }}t} \right)} \right),n \le \lambda .} \end{array}} \right.$$

First, note that if the satiation is above the threshold *λ*, no advertisement is given; hence, the score is 0. Second, the use of the agent’s future state in Eq. (7) allows for three types of outcomes for the score. The score will be positive if the activity increases the current value of the satiation; the score will be negative if the activity decreases the current value of the satiation; and the score will be zero if the activity does not change the current value of the satiation. In our framework, the agent chooses the activity with the highest positive score. If no activity fits that criteria, the agent “decides” to be idle (i.e., the agent does not perform any of the activities available in the ABM).

In general, once an activity is selected, the agent does not do anything else until the activity is completed. However, there are cases when it is useful for an agent to have the ability to interrupt a current activity in order to address a more pressing need. An example of this behavior is an agent stopping work in order to eat lunch. In the current model, the only activity that is interruptible is the work activity. The only activity that may interrupt working is eating lunch.

**Fig. 1:**
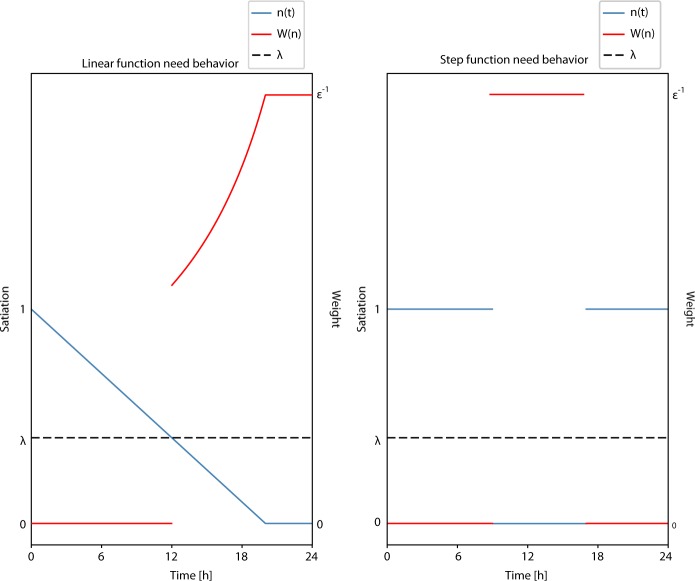
Decay behavior of needs. (Left) The behavior of a need modeled by a linear function. (Right) The behavior of a need modeled by a step function

Figure 1 displays a representation of the interaction of satiation, weight function, and threshold for the two mathematical behaviors of needs.

### Modeling the decision-making process for choosing behaviors

The following describes how the previously discussed methodology fits within the application of the ABM. The ABM initializes by assigning the agent’s fixed characteristics. This is necessary because many needs are dependent on the fixed characteristics of the agent. After initiation, the model moves an individual through time (1 minute at a time) by assigning the agent’s actions based on the agent’s needs over the simulation period.

**Fig. 2:**
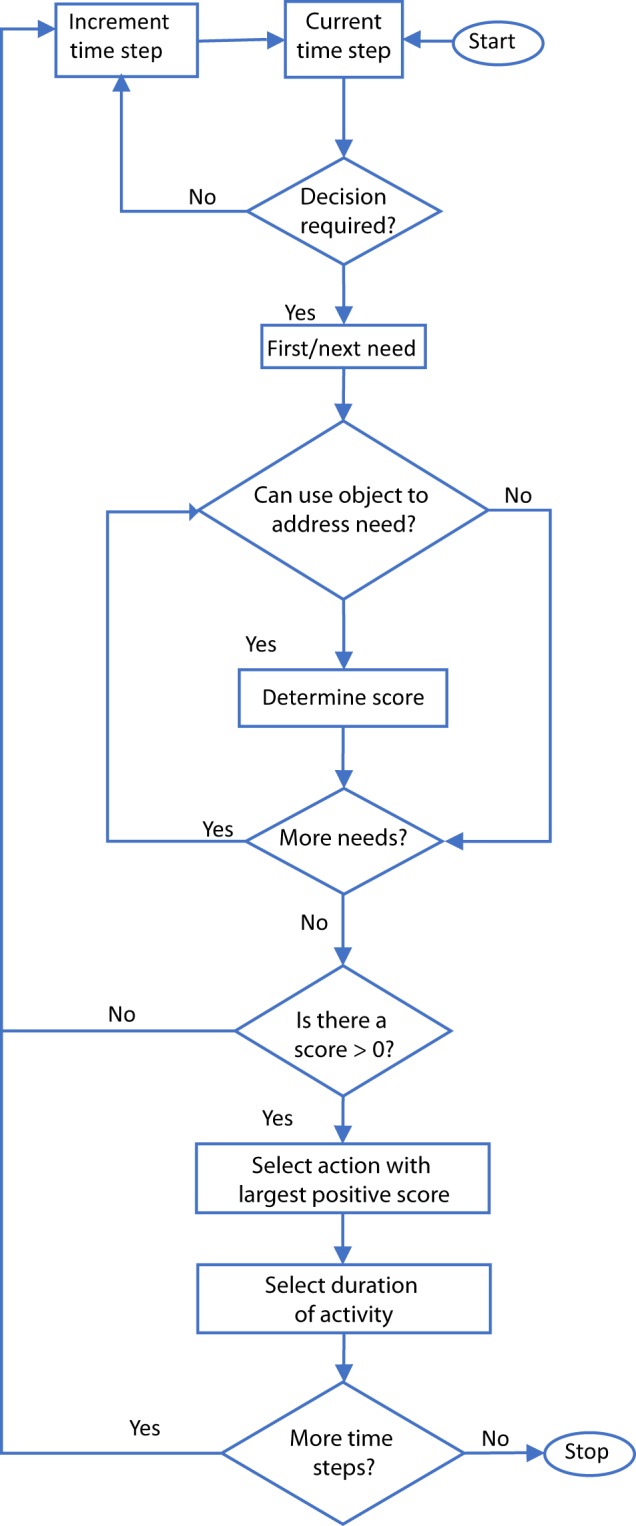
AI for action decision-making

Figure 2 illustrates the needs-based AI implemented in the ABM. At each time step, the AI allows the agent to go through the following decision-making process. First, the model begins by determining if the agent’s current status permits decision-making. We assume that the activities sleeping, commuting, and eating cannot be interrupted. During such activities, the agent has no desire to consider other activities. If the agent is able to make a decision to start a new activity (i.e. the agent is currently idle or performing a behavior that can be interrupted), the model assesses each of the agent’s needs. If a given need is unsatisfied, the model then determines if the respective need can be satisfied in the agent’s current environment. In order to make this determination, the objects in the environment communicate their availability and usefulness with respect to the given need to the agent. In communicating with an agent, an object advertises all of its available abilities that address the given need. For the given activities, the score of performing the activity is determined by using Eq. (7). Once all of the objects advertise their scores for the respective need, the scores relevant for the next need are addressed. Finally, the model determines which need has the highest positive score, and the corresponding activity is started. If the agent has no needs below threshold that can be met (i.e., the agent is not yet unsatisfied) or has no score greater than 0 associated with it (i.e., there are no available activities within the environment that may increase the satiation of the agent), the agent remains idle until the next time step. The simulation continues until the total prescribed time for the simulation has elapsed.

### Creating a model of variation in longitudinal activity patterns

We now describe the implementation of the above components and methods to create an algorithm to model individuals’ behaviors over time. We call this algorithm the Agent-Based Model of Human Activity Patterns (ABMHAP). Currently, this model only includes four behaviors (sleeping, eating, commuting, and working). These four behaviors break down into seven distinct activities: sleeping, eating breakfast, commuting to work, working, eating lunch, commuting from work, and eating dinner. Every day the agent is expected to sleep and eat breakfast, lunch, and dinner. The agent is expected to commute and work 5 days a week (Monday–Friday). Eating lunch is modeled as being able to interrupt working. Commuting to and from work are linked to working. The morning commute ends with the beginning of work, and the evening commute starts with the end of work. All activities continue until the need is fully sated. The value of *η* for commuting is set very low, resulting in a higher priority for leaving for work than for eating breakfast.

In the model, there are three environments: residence, workplace, and outdoors. The agent may move to or from the residence and outdoors and to or from the workplace and outdoors. The agent may not move from the residence directly to the workplace. In the residence, the objects required for sleeping and eating are present. In the workplace there are objects that allow working and eating. In the outside environment there is an object that allows movement to and from the workplace and residence.

### Modeling the decision-making process

In ABMHAP, we denote the set of parameters start time, end time, and duration as the *activity parameters* for a given activity. Let *T*_start_, *T*_end_, and Δ*T* be random variables that describe the start time, end time, and duration, respectively, for a given activity. And let *t*_start_, *t*_end_, and Δ*t* be realizations of the random variables *T*_start_, *T*_end_, and Δ*T*, respectively.

While each activity in the ABM has a start time, end time, and duration, the model defines two of the parameters for each non-linked activity. Activities that have a start time or an end time that are linked to another activity only require a duration. Table 2 specifies ABMHAP’s parameterization for each activity where X indicates that the activity parameter is directly parameterized.

**Table 2:** Display of parameters that define each activity

Activity	Start time	End time	Duration
Sleep	X	X	
Eat breakfast	X		X
Commute to work			X
Work	X	X	
Eat lunch	X		X
Commute from work			X
Eat dinner	X		X

X indicates which activity parameters are parameterized in the model

The sleep activity interacts with *Rest*’s satiation *n(t)* when the agent starts and ends the activity. Assume that at time *t*_0_, the satiation for *Rest n*(*t*_0_) ≤ *λ*; and the agent decides to initiate the sleep activity. When the agent starts the activity, the algorithm uses Eq. (3) to calculate the recovery rate *m*_recover_ and Eq. (4) to calculate Δ*t*_activity_. Recall that if *n*(*t*_0_) < *λ*, then the agent will sleep longer than the minimum recovery time (i.e., Δ*t*_activity_ > Δ*t*_recover_). At time Δ*t*_end_ = Δ*t*_start_ + Δ*t*_activity_, the agent ends the activity with *Rest* fully satisfied. The time for the next sleep occurrence is determined by the time that the value of *Rest*’s satiation *n*(*t*_0_) = *λ*.

The “eat breakfast”, “eat lunch”, and “eat dinner” activities interact with *Hunger*’s satiation *n*(*t*) when the agent starts and ends the respective activity. What follows is an analysis for the eat breakfast activity; the eat lunch and eat dinner activities behave similarly.

Assume that at time *t*_0_, *Hunger*’s satiation *n*(*t*_0_) ≤ *λ*; and the agent decides to initiate the eat breakfast activity. Assume that the agent has the values *t*_start_ and Δ*t* (see Table 2). When the agent starts the activity, the algorithm uses Eq. (3) to calculate the recovery rate *m*_recover_ by using Δ*t*_recover_ = Δ*t*. The true duration of the activity is calculated from Eq. (4). At time *t*_1_ = *t*_0_ + Δ*t*_activity_, the agent ends the activity with *Hunger* fully satisfied. A new set of values is sampled for *t*_*start*_ and Δ*t*. Finally, the satiation decay rate *m*_decay_ is calculated from Eq. (1) where Δ*t*_decay_ is the amount of time until the next meal starts (in this case of breakfast, the next meal is lunch).

The commute to or from work activity interacts with *Travel’s* satiation *n*(*t*) when the agent starts and ends the respective activity. What follows is an analysis for the commute to work activity; the commute from work activity behaves similarly.

Assume that at time *t*_0_, *Travel’s* satiation *n*(*t*_0_) = *η* ≤ *λ*; and the agent decides to initiate the commute activity. Assume that the agent has the value Δ*t* (see Table 2). When the agent starts the commute to work activity, the agent stays in the current activity until time *t* = *t*_0_ + Δ*t*. At this time, the agent ends the activity with *Travel* being fully satisfied. Finally, a new value is determined for Δ*t* for the next commute to work activity occurrence.

The work activity interacts with *Income’s* satiation *n*(*t*) when the agent starts and ends the respective activity. Assume that at time *t*_0_, *Income’s* satiation *n*(*t*_0_) = *η* ≤ *λ*; and the agent decides to initiate the work activity. Assume that the agent has the values *t*_start_ and *t*_end_ (see Table 2). When the agent starts the work activity, the agent stays in the current activity until time *t* = *t*_end_. The agent ends the activity with *Income* fully satisfied. Finally, a new set of values is sampled for *t*_start_ and *t*_end_ for the next work activity occurrence.

Competing needs occur at several points in a day. First, when the agent wakes it has three possible activities: remain idle, eat breakfast, or commute to work. If breakfast is selected, upon its termination the agent may decide to be idle or commute to work. Commuting and work are linked activities. Thus the agent must start work after the morning commute is over and commute home after the workday is complete. During work the agent may continue to work or break for lunch. Upon arrival at home the agent may be idle, eat dinner, or go to sleep.

Table 3 illustrates an example output of 1 day’s worth of an agent’s activity diary.

**Table 3:** Example activity diary for a working adult

Day	Start time	End time	Duration	Activity	Weekday	Environment
0	23:00	08:00	09:00	Sleep	Sunday	Residence
1	08:00	08:15	00:15	Eat breakfast	Monday	Residence
1	08:15	09:00	00:45	Commute to work	Monday	Outdoors
1	09:00	12:00	03:00	Work	Monday	Workplace
1	12:00	12:30	00:30	Eat lunch	Monday	Workplace
1	12:30	17:00	04:30	Work	Monday	Workplace
1	17:00	17:30	00:30	Commute from work	Monday	Outdoors
1	17:30	20:00	02:30	Idle	Monday	Residence
1	20:00	20:45	00:45	Eat dinner	Monday	Residence
1	20:45	23:30	02:45	Idle	Monday	Residence
1	23:30	08:00	08:30	Sleep	Monday	Residence
2	08:00	08:20	00:20	Eat breakfast	Tuesday	Residence

Time is represented as hours:minutes

### Parameterizing the longitudinal behavior

Each activity parameter *X* in Table 2 is modeled as belonging to its own truncated normal distribution (with width equal to one standard deviation) with mean *μ*, and standard deviation *σ*, respectively. The values of *μ* represents an agent’s long-term average behavior, and *σ* represents an agent’s day-to-day variation for the given activity parameter. Whenever an agent performs an activity, the activity parameters corresponding to the respective activity are sampled from the distributions defined by their values of *μ* and *σ*. The sampled values for the activity parameters in Table 2 are then used to determine the parameters in Eqs. (1)–(5) in order to determine the satiation levels for the individual’s needs. This approach to parameterization is used to facilitate the use of existing survey data on human behaviors for calibrating ABMHAP.

### Code availability

ABMHAP is coded in Python 3.5 [17]. More information about ABMHAP is found in the Supplementary Information.

## Results

We now present a case study of the above methodology as it is implemented in ABMHAP. The exact values for *μ* and *σ* for each activity parameter used in the example simulation are presented in Table 4. These values are chosen for demonstrative purposes.

**Table 4:** Numerical values used in ABMHAP example analysis

Activity	Start time [hours]	End time [hours]	Duration [hours]
Sleep	*μ* = 22 *σ* = 0.5	*μ* = 8 *σ* = 2.5	
Eat breakfast	*μ* = 8.25 *σ* = 0		*μ* = 0.25 *σ* = 0
Commute to work			*μ* = 0.5 *σ* = 0
Work	*μ* = 9 *σ* = 0	*μ* = 17 *σ* = 0.08	
Eat lunch	*μ* = 12 *σ* = 0.25		*μ* = 0.5 *σ* = 0.17
Commute from work			*μ* = 1 *σ* = 0
Eat dinner	*μ* = 19 *σ* = 0.17		*μ* = 0.75 *σ* = 0.08

**Fig. 3:**
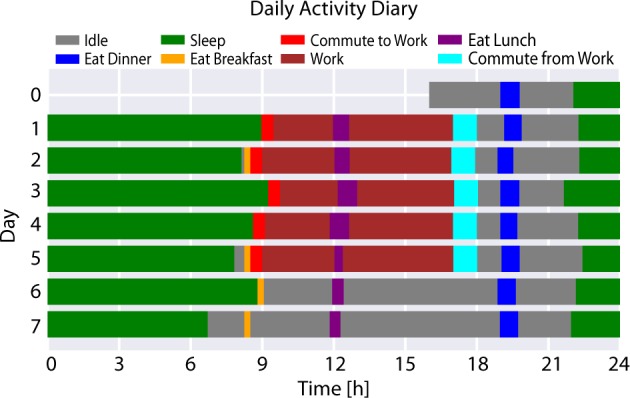
Visualization of ABMHAP simulation output

ABMHAP was used to model one individual for a period of 7 days and 8 h. The simulation started on Day 0, Sunday, at 16:00 with the agent in the residence environment and ended on Day 8, Monday, at 0:00. (The extra 8 h on day zero are required to properly set up the modeling of the initial sleep event on Day 0.) The results of the simulation, which can be called an “activity diary,” are visualized in Fig. 3.

**Fig. 4:**
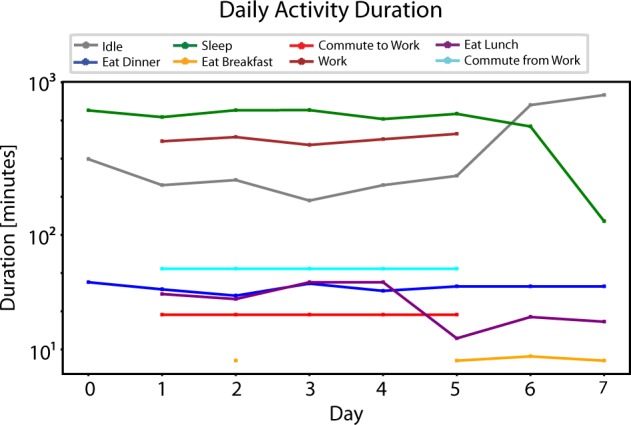
Visualization of activity durations of an ABMHAP simulation. The durations are expressed in a log_10_ scale

The simulation shows that ABMHAP is capable of producing longitudinal behavior patterns of a working adult that include day-to-day variation. Here, we have an agent who attempts to sleep around 22:00, wake around 8:00, eat breakfast around 8:15 for about 15 min, eat lunch around 12:00 for about 30 min, eat dinner around 19:00 for about 45 min, work approximately from 9:00–17:00, commute to work for 30 min, and commute from work for 60 min. In addition, ABMHAP captures the difference in behavior from workdays (Monday–Friday) and non-workdays (Saturday and Sunday), notably the absence of working and commuting on the weekends. The model is able to simulate day-to-day variation in the activities by having *σ≠*0 for the respective activity parameters. Figure 4 portrays the daily variation of activity duration. Without this variation, the schedule for each weekday or weekend would be exactly the same, which is unrealistic. People do not repeat their actions exactly from 1 day to the next.

**Fig. 5:**
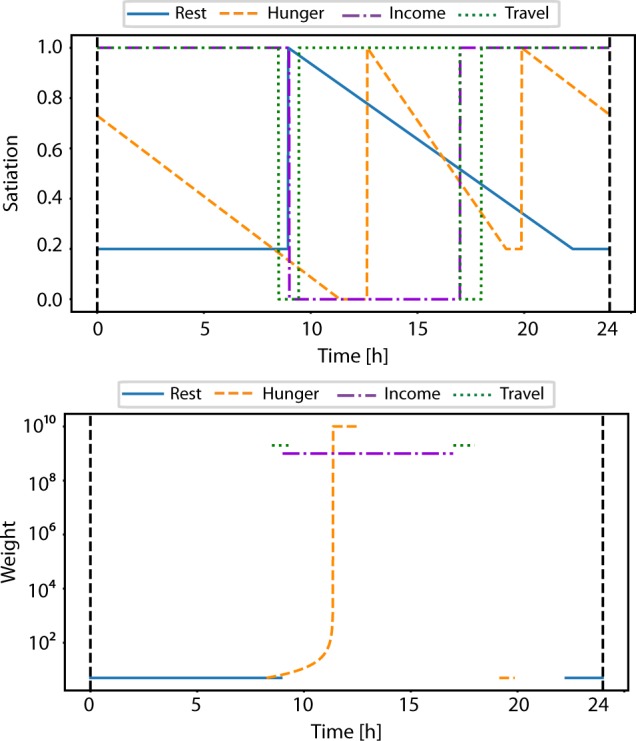
The mathematical components involved for decision-making from the ABMHAP simulation on Day 1, Monday. (Top) The satiation values *n*(*t*) for each need (recall the value of the threshold *λ* = 0.2). (Bottom) The non-zero values of the weight function *W*(*n*). The values are expressed in a log_10_ scale

In this example, we deliberately assign a large value of *σ* in sleep end time to show how ABMHAP may handle competing needs in the morning. Figure 3 shows that on Days 1, 3, and 4, the large value of *σ* in sleep end time causes the agent to wake up so sufficiently late that it causes the agent to skip breakfast. In these cases when waking up, the agent has competing unsatisfied needs *Hunger* and *Travel* (and in the case of Day 3, *Income*) to consider. Currently, the agent is parametrized to favor commuting to work rather than eating breakfast, which mimics how people may rush to work in order to minimize the amount of time that they may be late. The agent achieves this behavior by having *Travel’s* satiation be set to a much lower value than *Hunger’s*. This can be seen in Fig. 5, which shows how both the satiation values and the weight function values change over time in Day 1 for each need. On Day 2 and Day 5, the agent wakes up early enough that there is idle time between working and eating breakfast, causing the agent to eat breakfast and commute to work on time. On Day 6, the agent wakes up far after the expected time to eat breakfast. However, since the only unsatisfied need is *Hunger* at this point in time, the agent simply starts eating breakfast. Lastly on Day 7, the agent wakes up earlier than expected, and each need is addressed as soon as it becomes unsatisfied.

## Discussion

The ability to simulate longitudinal patterns in exposure-related behavior meet a critical need in the field of exposure assessment. It is difficult to perform longitudinal studies of exposure behaviors, and only a limited number of such studies are available. Unfortunately, the determination of the safety of chemicals requires information on the longitudinal patterns of exposure [18] which in turn require data on longitudinal patterns of human behavior [19]. The presented framework (and its implementation in ABHMAP) is proposed as a method for generating data that avoids the problems of measuring longitudinal patterns. This framework allows scientists to use behavior data on the frequency and duration of various types of activities that are collected in isolation and generate longitudinal behavior patterns (longitudinal activity diaries) as a coupled system of activities.

The current version of ABMHAP only addresses four types of behavior and does not address the more complex needs that are based on human interactions (child rearing, family activities, unscheduled group activities) nor does it address instances when demands of jobs or education supersede meals or sleep (working or studying late). However, the framework can be readily extended to consider these behaviors. The concepts defined in the framework (i.e., agents, objects that satisfy needs, environments, linked activities) and the proposed AI together present an approach that can be extended to any number of behaviors. The equations used to describe needs, weights, and scores are sufficiently flexible to allow the modeling of an individual’s prioritization of competing needs over time. The parameter values in the equations can be informed by first principle arguments or calibrated with data from empirical surveys.

Exposures resulting from consumer product use can also be addressed by the framework. Consumer products are used as a result of individuals having needs that the products satisfy. Thus, the needs-based approach can be applied to the use of these products. Models that combine the four needs in ABMHAP and the consumer product-specific needs may be especially helpful in identifying times and durations when exposure events may occur. For example, many behaviors related to the use of consumer products that result in exposure events (e.g. cleaning kitchen floors with floor cleaner) would occur when an agent is present in the residence but not doing any of the four behaviors: sleeping, eating, working, and commuting. Use of other products, however, may happen concurrently with one of the four behaviors (e.g. putting on hand lotion while at work). In addition, ABMHAP’s ability to generate activity patterns provides a unique opportunity for modeling interactions with many chemical sources (pollutants in ambient and indoor air) since it defines when an individual is in the workplace, outdoors, or at home.

### Summary and future Work

The results suggest that needs-based AI underlying ABMHAP may be a suitable way of simulating the actions of working adults living in single-occupancy residences for the investigated behaviors. The model can incorporate data on day-to-day variation in behaviors and capture interactions between an individual’s behaviors. As a next step in our research, we plan on calibrating ABMHAP with longitudinal survey data from CHAD and making longitudinal predictions of the four behaviors in adults and children. We do acknowledge that the case study only addresses a limited number of actions. In the future, this model could be extended to include behaviors involving the use of consumer products in order to help exposure-assessors identify times when these exposure-events may occur.

### Disclaimer

The views expressed in this article are those of the authors and do not necessarily represent the views or policies of the U.S. Environmental Protection Agency.

## Electronic supplementary material

Supplemental Material
