# Supplementary material for: Simulating exposure-related behaviors using agent-based models embedded with needs-based artificial intelligence
Source: J Expo Sci Environ Epidemiol. 2018 Sep 21;30(1):184–93. doi: 10.1038/s41370-018-0052-y (PMC6914672; doi:10.1038/s41370-018-0052-y)
Supplement: Supplementary file 1 — Supplemental Material [file 41370_2018_52_MOESM1_ESM.pdf]

The Agent-Based Model of Human Activity Patterns (ABMHAP): Documentation and Users Guide

Release 2017.08

Namdi Brandon

August 14, 2017

Contents

1 How to Run the Code **3**

1.1 Setting the input parameters 3
1.2 Running the simulation 6
1.3 Interpreting the output 7

2 Source Directory **9**

2.1 activity module 9
2.2 asset module 11
2.3 bed module 12
2.4 bio module 12
2.5 commute module 14
2.6 diary module 18
2.7 eat module 21
2.8 food module 25
2.9 home module 26
2.10 hunger module 27
2.11 income module 30
2.12 interrupt module 31
2.13 interruption module 32
2.14 location module 33
2.15 meal module 34
2.16 my_globals module 36
2.17 need module 39
2.18 occupation module 40
2.19 params module 46
2.20 person module 51
2.21 rest module 53
2.22 scheduler module 56
2.23 sleep module 57
2.24 social module 59
2.25 state module 62
2.26 temporal module 64
2.27 transport module 67
2.28 travel module 67
2.29 universe module 68
2.30 work module 72
2.31 workplace module 74

3 Run Directory **75**

3.1 main module . . . 75

3.2 main_params module . . . 76

3.3 scenario module . . . 76

3.4 singleton module . . . 78

4 Indices and tables **81**

Python Module Index **83**

The Agent-Based Model of Human Activity Patterns (ABMHAP, pronounced "ab-map") is one of the modules for the Life Cycle Human Exposure Model (LC-HEM) project as described in the United States Environmental Protection Agency (U.S. EPA) research plan, which may be found here. ABMHAP is a model capable of creating agents that simulate longitudinal human behavior. The current version of ABMHAP is able to simulate daily routines for the following behaviors:

1. Sleeping
2. Eating Breakfast
3. Eating Lunch
4. Eating Dinner
5. Working
6. Commuting to Work
7. Commuting from Work
8. Being idle (i.e., time spent not doing the above activities)

The current version of ABMHAP requires the user to input parameters that describe the longitudinal variation in behavior of a single individual.

The current version of ABMHAP is written in Python version 3.5.3. More information on the Python programming language may be found here. The Python libraries that must be installed in order for ABMHAP to run are listed below.

- matplotlib
- multiprocessing
- numpy
- pandas
- scipy
- sphinx
- statsmodels

ABMHAP is written by Dr. Namdi Brandon (ORCID: 0000-0001-7050-1538).

Disclaimer The United States Environmental Protection Agency through its Office of Research and Development has developed this software. The code is made publicly available to better communicate the research. All input data used for a given application should be reviewed by the researcher so that the model results are based on appropriate data for any given application. This model is under continued development. The model and data included herein do not represent and should not be construed to represent any Agency determination or policy.

CHAPTER 1

How to Run the Code

The following describes how to run an ABMHAP simulation of one agent. In order to do so, the user must do the following:

1. set the input parameters of the simulation in the file `\run\main_params.py`
2. run the executable using `\run\main.py`

1.1 Setting the input parameters

In order to run ABMHAP, the user must set the following types of input parameters in `\run\main_params.py`:

1. input parameters that govern the general logistics of the simulation
2. input parameters that govern the the length of simulation duration
3. input parameters that define the behavior of the agent

For illustrative purposes, what follows is a demonstration of how to parametrize a run for ABMHAP.

The below code does the following:

- informs the algorithm to not print the output to the screen
- informs the algorithm to not plot the output
- informs the algorithm to not save the output to a file
- should the output file be saved, sets the output file to `\some_directory\outputfile.csv`

The user must set the input parameters that govern the general logistics of the simulation:

```
# whether (if True) or not (if False) the output of the simulation should
# print a message to screen
do_print    = False

# whether (if True) or not (if False) the output of the simulation should
# be plotted a message to screen
do_plot     = False

# whether (if True) or not (if False) the output of the simulation should
# be saved in a file
do_save     = False

# the name of the output file should the output be saved. The filename
```

```
# should have a ".csv" extension
fname        = 'some_directory\\outputfile.csv'
```

The following code shows how to set ABMHAP to run starting on Sunday, Day 0 starting from 16:00 and ending on Monday, Day 7 at 0:00. It's recommended that the user start running the code on a Sunday or Saturday at 16:00 in order to minimize potential activity conflicts at initiation.

The user must set the input parameters dealing with the duration of the simulation:

```
# the number of days for the simulation
num_days     = 7

# the number of additional hours
num_hours    = 8

# the number of additional minutes
num_min      = 0
```

The user must set the input parameters dealing with when in the simulation year the simulation should start:

```
# start the simulation on Sunday, Day 0 at 16:00
t_start      = WINTER * SEASON_2_MIN + 0 * WEEK_2_MIN \
             + SUNDAY * DAY_2_MIN + 16 * HOUR_2_MIN
```

where the following constants are useful in assigning input parameters that define the start time of the simulation:

```
# an agent-based model module with capabilities concerning time
import temporal

# the value of Sunday
SUNDAY          = temporal.SUNDAY

# convert one day into minutes
DAY_2_MIN       = temporal.DAY_2_MIN

# convert one hour into minutes
HOUR_2_MIN      = temporal.HOUR_2_MIN

# the number of minutes in one season (13 weeks)
SEASON_2_MIN    = temporal.SEASON_2_MIN

# the number of minutes in one week
WEEK_2_MIN      = WEEK_2_MIN

# the winter season (has the value 0)
WINTER          = temporal.WINTER
```

The user must set the input parameters that govern the behavior of the agent. The input parameters will govern the agent's behavior for the following activities.

1. sleeping
2. eating breakfast
3. eating lunch
4. eating dinner
5. working
6. commuting to work

7. commuting from work

In order to set the sleeping behavior, the user must set the the mean and standard deviation for the start time and end time for the sleep activity. The agent's behavior for sleeping is set as follows:

```
# set the mean start time to be 22:00
sleep_start_mean     = np.array( [22 * HOUR_2_MIN] )

# set the standard deviation of the start time to be 30 minutes
sleep_start_std      = np.array( [30] )

# set the mean end time to be 8:00
sleep_end_mean       = np.array( [8 * HOUR_2_MIN] )

# set the standard deviation of the end time to be 15 minutes
sleep_end_std        = np.array( [15] )
```

In order to set the eat breakfast behavior, the user must set the mean and standard deviation for the start time and duration for the eat breakfast activity. The agent's behavior for eating breakfast is set as follows:

```
# set the mean start time to be 8:00
bf_start_mean       = np.array( [8 * HOUR_2_MIN] )

# set the standard deviation of the start time to be 10 minutes
bf_start_std        = np.array( [10] )

# set the mean duration to be 15 minutes
bf_dt_mean          = np.array( [15] )

# set the standard deviation of the duration to be 10 minutes
bf_dt_std           = np.array( [10] )
```

In order to set the eat lunch behavior, the user must set the mean and standard deviation for the start time and duration for the eat lunch activity. The agent's behavior for eating lunch is set as follows:

```
# set the mean start time to be 12:000
lunch_start_mean      = np.array( [12 * HOUR_2_MIN] )

# set the standard deviation of start time to be 15 minutes
lunch_start_std       = np.array( [15] )

# set the mean duration to be 30 minutes
lunch_dt_mean         = np.array( [30] )

# set the standard deviation of duration to be 10 minutes
lunch_dt_std          = np.array( [10] )
```

In order to set the eat dinner behavior, the user must set the mean and standard deviation for the start time and duration for the eat dinner activity. The agent's behavior for eating dinner is set as follows:

```
# set the mean start time to be 19:00
dinner_start_mean       = np.array( [19 * HOUR_2_MIN] )

# set the standard deviation of start time to be 10 minutes
dinner_start_std        = np.array( [10] )

# set the mean of duration to be 45 minutes
dinner_dt_mean          = np.array( [45] )
```

```
# set the standard deviation of duration to be 5 minutes
dinner_dt_std             = np.array( [5] )
```

In order to set the work behavior, the user must set the mean and standard deviation for the start time and end time for the work activity. The agent's behavior for working is set as follows:

```
# set the mean start time to be 9:00
work_start_mean      = np.array( [9 * HOUR_2_MIN] )

# set the standard deviation of start time to be 15 minutes
work_start_std       = np.array( [15] )

# set the mean end time to be 17:00
work_end_mean        = np.array( [17 * HOUR_2_MIN] )

# set the standard deviation of end time to be 5 minutes
work_end_std         = np.array( [5] )
```

The user must set the agent's employment status. The agent's employment status is set as follows:

```
# an agent-based model module for functionality dealing with occupation
import occupation

# set the job identifier (job id) as standard job if the agent
# is supposed to work
job_id    = occupation.STANDARD_JOB

# OR set the job identifier (job id) as not having a job if the agent
# is NOT supposed to work
job_id    = occupation.NO_JOB
```

In order to set the commute to work behavior, the user must set the mean and standard deviation for the duration of the commute to work activity. The agent's behavior for commuting to work is set as follows:

```
# set the mean duration to be 30 minutes
commute_to_work_dt_mean      = np.array( [30] )

# set the standard deviation to be 10 minutes
commute_to_work_dt_std       = np.array( [10] )
```

In order to set the commute from work behavior, the user must set the mean and standard deviation for the duration of the commute from work activity. The agent's behavior for commuting from work is set as follows:

```
# set the mean duration to be 30 minutes
commute_from_work_dt_mean      = np.array( [30] )

# set the standard deviation to be 10 minutes
commute_from_work_dt_std       = np.array( [10] )
```

1.2 Running the simulation

After defining all of the parameters in the file `\run\main_params.py`, the code is run by doing the following:

1. go to the `\run` directory.
2. enter `python main.py` into the terminal (or command line)
3. press enter (or return)

1.3 Interpreting the output

ABMHAP outputs the record of the activities that the agent did during the simulation. This record is called an **activity diary**. An activity diary is a chronological record contains the following information about each activity: day, start time, end time, duration, and location.

Below is an example of the output of ABMHAP. Recall that ABMHAP saves the activity diary in terms of a .csv file

day	start	end	dt	act	loc
0	16	19	3	-1	0
0	19	19.75	0.75	4	0
0	19.75	22	2.25	-1	0
0	22	8	10	6	0
1	8	8.25	0.25	3	0
1	8.25	8.5	0.25	-1	0
1	8.5	9	0.5	2	1
1	9	12	3	7	3
1	12	12.5	0.5	5	3
1	12.5	17	4.5	7	3
1	17	17.5	0.5	1	1
1	17.5	19	1.5	-1	0
1	19	19.75	0.75	4	0
1	19.75	22	2.25	-1	0
1	22	8	10	6	0

where day, start, end, dt, act, and loc represent the day the activity starts, the start time of the activity (in hours), the end time of the activity (in hours), the duration of the activity (in hours), the activity identifier, and the location identifier, respectively. In the results, the time of day 16:30 is represented as 16.5.

The following table is an interpretation of the example output shown above. In the table, the duration is expressed in minutes.

Day	Start	End	Duration	Activity Code	Location Code
0	16:00	19:00	180	Idle	Home
0	19:00	19:45	45	Eat dinner	Home
0	19:45	22:00	135	Idle	Home
0	22:00	8:00	600	Sleep	Home
1	8:00	8:15	15	Eat breakfast	Home
1	8:15	8:30	15	Idle	Home
1	8:30	9:00	30	Commute to work	Out of doors
1	9:00	12:00	180	Work	Workplace
1	12:00	12:30	30	Eat lunch	Workplace
1	12:30	17:00	270	Work	Workplace
1	17:00	17:30	30	Commute from work	Out of doors
1	17:30	19:00	90	Idle	Home
1	19:00	19:45	45	Eat dinner	Home
1	19.45	22:00	135	Idle	Home
1	22:00	8:00	600	Sleep	Home

The activity codes map as the following:

Activity	Activity Code
Idle	-1
Commute from work	1
Commute to work	2
Eat breakfast	3
Eat dinner	4
Eat lunch	5
Sleep	6
Work	7

The location codes map as the following:

Location	Location Code
Home	0
Out of doors	1
Workplace	3

CHAPTER 2

Source Directory

These files are the key modules that are used to create the ABMHAP algorithm.

Contents:

2.1 activity module

This module contains code that governs the activities that the agent performs in order to satisfy its needs.

This module contains the following class: *activity.Activity*.

Module author: Dr. Namdi Brandon

class activity.**Activity**

Bases: object

An activity enables a *person.Person* to address its satiation $n(t)$. This class's purpose is to encapsulate general capabilities that specific instances of activity will derive from.

Variables

- **category** (*int*) – an unique identifier naming the type of activity.
- **t_end** (*int*) – the end time of the activity [universal time, seconds]
- **t_start** (*int*) – the start time of the activity [universal time, seconds]
- **dt** (*int*) – the duration of the activity [seconds]

advertise (*the_need*, *dt*)

Calculates the advertised score of doing an activity. Let Ω be the set of all needs addressed by the activity. The score is calculated by doing the following

$$S = \begin{cases} 0 & n(t) > \lambda \\ \sum_{j \in \Omega} W_j(n_j(t)) - W_j(n_j(t + \Delta t)) & n(t) \leq \lambda \end{cases}$$

where $W(n)$ is the weight function for the particular need.

Parameters

- **the_need** (need.Need) – the primary need associated with the respective activity
- **dt** (*int*) – the duration Δt of doing the activity [seconds]

Returns score the score of the advertisement

Return type float

advertise_interruption()

Advertise the score if this activity interrupts another activity.

Note: This function should be overloaded in derived classes.

Returns score the advertised score

Return type float

end(*p*)

This function handles some of the common logistics in ending a specific activity assuming the activity ends without interruption.

Currently the function does the following:

1. Reset the *state.State* variable's start time to the current time
2. Reset the *state.State* variable's end time to the current time

Parameters **p** (person.Person) – the person whose activity is ending.

Returns None

halt(*p*)

This function handles some of the common logistics in ending a specific activity due to an interruption.

Currently the function does the following:

1. Reset the *state.State* variable's start time to the current time
2. Reset the *state.State* variable's end time to the current time

Parameters **p** (person.Person) – The person whose activity is being interrupted.

Returns None

print_id()

This function represents the activity category as a string.

Return msg The string representation of the category

Return type str

start()

This function starts a specific activity.

Note: This function is meant to be overloaded by derived activity classes.

Returns None

toString()

This function represents the activity object as a string.

Return msg The string representation of the activity object

Return type str

2.2 asset module

This module contains code that governs objects that enable access to activities (`activity.Activity`) that an agent may use in order to address a need.

This module contains the following class: `asset.Asset`.

Module author: Dr. Namdi Brandon

class `asset.`**`Asset`**

Bases: `object`

An asset is an object that allows the agent to perform an activity. Each asset contains a list Activities that an agent can use to perform actions.

Variables

- **`activities`** (*dict*) – This is a dictionary of all the activities associated with this asset.
- **`category`** (*int*) – This indicates the type of asset.
- **`id`** (*int*) – This is an identifier number for the asset.
- **`'location'`** (location.Location) – This is the location of the asset.
- **`max_users`** (*int*) – This indicates the maximum number of users that can simultaneously access the asset.
- **`num_users`** (*int*) – This is the current number of users for the asset.
- **`status`** (*int*) – This is the state of the asset.

`free`()

This function changes the state of an asset once it is freed by a Person by doing the following:

1.Decreases the number of users of the asset by 1

2.If the number of users is zero, the status of the asset is set to idle (`state.IDLE`)

Returns None

`initialize`(*people*)

This function initializes the asset at the beginning of the simulation.

Note: This function is meant to be overridden

Parameters **`people`** (*list[person.Person]*) – the Person objects who could be using the asset.

Returns None

`print_category`()

This function represents the category as a string.

Returns the string representation of the category

Return type str

`reset`()

This function does the following.

1.Sets the number of users to zero

2. Sets the asset's status to idle

Returns None

toString()

This function represents the asset as a string.

Return msg The string representation of the asset object.

Return type str

update()

This function changes the state of the asset once it is used by a Person.

1. Increases the number of people by 1
2. If the number of users is at the maximum number, set the asset's status to busy
3. If the number of users is less than the maximum number, set the assets' status to busy but able to be used by another agent

Returns None

2.3 bed module

This module contains code that enables the agent to use a bed. This class allows access to the sleep (*sleep.Sleep*) activity.

This module contains the following class: *bed.Bed*.

Module author: Dr. Namdi Brandon

class bed.**Bed**

Bases: *asset.Asset*

This asset models a bed. It allows the agent to address the Rest (*rest.Rest*) need by doing the sleep (*sleep.Sleep*) action .

2.4 bio module

This module contains information about a *person.Person*'s biology.

This module contains the following class: *bio.Bio*.

Module author: Dr. Namdi Brandon

class bio.**Bio**

Bases: object

This class holds the biologically relevant information for a person. This information is:

- Age
- Gender
- Mean / standard deviation of start time for sleeping
- Mean / standard deviation of end time for sleeping

•Probability distribution function sleep start time / end time

Variables

- **age** (*int*) – the age [years]
- **gender** (*int*) – the gender
- **sleep_dt** (*int*) – the duration of time for a sleep event [minutes]
- **sleep_start_mean** (*int*) – the mean start time for a sleep event [minutes]
- **sleep_start_std** (*int*) – the standard deviation for a starting a sleep event [minutes]
- **sleep_start** (*int*) – the start time for sleep [minutes, time of day]
- **sleep_start_univ** (*int*) – the start time for sleep[minutes, universal time]
- **sleep_end_mean** (*int*) – the mean end time for a sleep event [minutes]
- **sleep_end_std** (*int*) – the standard deviation for end time for a sleep event [minutes]
- **sleep_end** (*int*) – the end time for sleep[minutes, time of day]
- **sleep_end_univ** (*int*) – the end time for sleep [minutes, universal time]
- **start_trunc** (*int*) – the number of standard deviations to allow when sampling sleep start time truncated distribution
- **end_trunc** (*int*) – the number of standard deviations to allow when sampling sleep end time truncated distribution
- **f_sleep_start** (*func*) – the distribution data for start time for sleep
- **f_sleep_end** (*func*) – the distribution data for end time for sleep

calc_awake_duration(*t*)

This function calculates the amount of time the person is expected to be awake.

Parameters **t** (*int*) – time of day [minutes]

Returns the duration [minutes] until the agent is expected to awaken

print_gender()

This function returns a string representation of gender

Returns the string representation of gender

Return type str

set_sleep_params(*start_mean*, *start_std*, *end_mean*, *end_std*)

This function sets the biological sleep parameters themselves and the sleep parameter distribution functions.

Parameters

- **start_mean** (*int*) – the mean sleep start time [minutes]
- **start_std** (*int*) – the standard deviation of start time [minutes]
- **end_mean** (*int*) – the mean sleep end time [minutes]
- **end_std** (*int*) – the standard deviation of end time [minutes]

Returns None

toString(*do_decimal=False*)

This function represents the `bio.Bio` object as a string.

Parameters **do_decimal** (*bool*) – This controls whether or not to represent the values in time in a decimal (hours) format where [1:30pm is 13.5] if True or as the minutes in the day if False [1:30pm is 13 * 60 + 30].

Return msg the string representation of the Bio object

Return type string

update_sleep_dt()

This function sets the duration of sleep.

Returns None

update_sleep_end()

This function samples the sleep end time distribution and sets the end time.

Returns None

update_sleep_end_univ(*time_of_day*, *t_univ*)

This function sets the end time for sleep in terms of universal time.

Parameters

- **time_of_day** (*int*) – the current time of day [minutes]
- **t_univ** (*int*) – the universal time [minutes]

Returns None

update_sleep_start()

This function samples the sleep start time distribution and sets the start time.

Returns None

update_sleep_start_univ(*time_of_day*, *t_univ*)

This function sets the start time for sleep in terms of universal time.

Parameters

- **time_of_day** (*int*) – the current time of day [minutes]
- **t_univ** (*int*) – the universal time [minutes]

Returns None

update_time_univ(*time_of_day*, *t_univ*, *t*)

This function updates a time t, which represents sleep start time or end time, to be in the next occurrence

Parameters

- **time_of_day** (*int*) – the current time of day [minutes]
- **t_univ** (*int*) – the universal time [minutes]
- **t** (*int*) – the time to be set[minutes, time of day]

Return out the time of the next event in universal time

Return type int

2.5 commute module

This module contains about activities associated with commuting to and from work. This class is an *activity.Activity* that gives a *person.Person* the ability to commute to/ from work/ school and satisfy the need Travel *travel.Travel*.

This module contains the following classes:

1. *commute.Commute* (general commuting capability)
2. *commute.Commute_To_Work* (commute to work/ school)
3. *commute.Commute_From_Work* (commute from work/ school)

Module author: Dr. Namdi Brandon

class commute.**Commute**

Bases: *activity.Activity*

This class allows for commuting. This class is to be derived from.

end(*p*, *local*)

This function handles the end of an Activity.

Parameters

- **p** (person.Person) – the person of interest
- **local** (*int*) – the local location (work or home)

Returns None

end_commute(*p*)

This function ends the commuting activity.

Note: This function is to be overridden

Parameters **p** (person.Person) – the person of interest

Returns None

start(*p*)

This handles the start of the commute activity.

- If the current location of person is at home, the person is going to work, so set the location to `location.OFF_SITE`
- If the current location of the person is off site, the person is going back home, so set the location to `location.HOME`

Parameters **p** (person.Person) – the person of interest

Returns None

start_commute(*p*)

This function sets the variables pertaining to starting the commute activity.

1. set the status of the person to `location.TRANSIT`
2. set the location of the asset to `location.TRANSIT`
3. set the person’s state start time of the commute
4. set the person’s state end time for the commute
5. update the asset
6. update the scheduler for the travel need for the end of the commute

Parameters **p** (person.Person) – the person of interest

Returns None

class `commute.`**`Commute_From_Work`**

Bases: `commute.Commute`

This class allows for the activity: commuting from work.

`advertise`(*p*)

This function calculates the score of an activities advertisement advertise the score to commute.

1.calculate advertisement only if the person is located at work (off-site)

2.calculate the score

$$S = \begin{cases} 0 & n_{travel}(t) > \lambda \\ W(n_{travel}(t)) - W(n_{travel}(t + \Delta t)) & n_{travel}(t) \leq \lambda \end{cases}$$

Parameters **p** (person.Person) – the person of interest

Returns the advertised score

Return type float

`calc_end_time`(*p*)

1.calculate the end time (minutes, universal time) of the commute

2.set the the end time in the person's state

Parameters **p** (person.Person) – the person of interest

Returns None

`end`(*p*)

This function handles the end of an Activity.

Parameters **p** (person.Person) – the person of interest

Returns None

`end_commute`(*p*)

This function sets the variables pertaining to ending the commute activity.

1.Sets the person's state to idle(`state.IDLE`)

2.Updates the asset's state and number of users

3.Sets the travel magnitude

4.Sets the work magnitude to `need.MAG_WORK`, to allow for work to be the next activity, even if commute ends begin the work-start time

5.Sets the person's state's end time

Parameters

- **p** (person.Person) – person of interest
- **destination** (*int*) – a local location where the commute ends (home or workplace)

Returns None

start (*p*)
This handles the start of the commute activity.

If the current location of person is at home, the person is going to work, so set the location to `location.OFF_SITE`

If the current location of the person is off site, the person is going back home, so set the location to `location.HOME`

Parameters **p** (person.Person) – the person of interest

Returns None

class `commute.`**Commute_To_Work**
Bases: *commute.Commute*

This class allows for the activity: commute to work

advertise (*p*)
This function calculates the score of an activities advertisement advertise the score to commute.

1. calculate advertisement only if the person is located at work (off-site)

2. calculate the score

$$S = \begin{cases} 0 & n_{travel}(t) > \lambda \\ W(n_{travel}(t)) - W(n_{travel}(t + \Delta t)) & n_{travel}(t) \leq \lambda \end{cases}$$

Parameters **p** (person.Person) – the person of interest

Return score the advertisement score

Return type float

calc_end_time (*p*)
Given the commute duration, store the end time.

1. calculate the end time [universal time] of the commute.

2. store the end time in the person.state

Parameters **p** (person.Person) – the person of interest

Returns None

end (*p*)
This function handles the logistics of ending the commute to work activity.

Parameters **p** (person.Person) – the person of interest

Returns None

end_commute (*p*)
This function handles the logistics concerning ending the commute.

1. the asset is freed up from use

2. the magnitude of the travel need is set $n_{travel} = 1$

3. the person's state is set to idle (`state.IDLE`)

4. the person's location is set to the location of the job

5.the asset's location is set to the location of the job

6.the person's income need is set to $n_{income} = \eta_{work}$

7.update the commute to work duration

8.calculate the time until the next leave work event

9.update the schedule for the travel need

Parameters **p** (person.Person) – the person of interest

Returns

start (*p*)

This function handles the start of the commute to work activity. If the current location of person is at home, the person is going to work, so set the location to workplace location (`location.OFF_SITE`)

Parameters **p** (person.Person) – the person of interest

Returns None

start_commute (*p*)

This function sets the variables pertaining to starting the commute to work activity.

1.set the person's status to `state.TRANSIT`

2.set the asset's location to `location.TRANSIT`

3.set the person's state start time to the current time

4.calculate the end time of commute to work

5.update the asset's update

6.update the scheduler for the travel need to take into account the end of the commute

7.update the scheduler for the income need to take into account the end of the commute

Parameters **p** (person.Person) – the person of interest

Returns None

2.6 diary module

This module contains code that governs the activity-diaries. Each activity diary contains dataframes that store the activity-diaries for each person. The activity-diaries are the output of the Agent-Based Model of Human Activity Patterns (ABMHAP) simulation.

This module contains class *`diary.Diary`*.

Module author: Dr. Namdi Brandon

class `diary.`**Diary** (*t*, *act*, *local*)

Bases: `object`

This class represents the activity-diaries for a person.

Parameters

- **t** (*numpy.ndarray*) – the start times for each activity [universal time, minutes]
- **act** (*numpy.ndarray*) – the activity code done at each time step [integer] (flattened array)

- **local** (*numpy.ndarray*) – the history of location codes done by a person

Variables

- **colnames** (*list*) – the column names for the activity diary in order
- **df** (*pandas.core.frame.DataFrame*) – the activity-diary

create_activity_diary (*t*, *act*, *local*)

This function creates the activity diary for a given agent in the simulation.

The activity diary contains:

1. The start-time and end-time for each activity
2. The activity code

Parameters

- **t** (*numpy.ndarray*) – the simulation times [universal time, minutes]
- **act** (*numpy.ndarray*) – the activity code done at each time step [integer] (flattened array)

Returns a tuple containing the following: the array indices for each activity grouping, the activity diaries in a numerical format, the activity diary in a string format, and the column names for each data type

Each diary is a tuple that contains the following:

1. the day number of the start of the activity
2. the (start-time, end-time) for the activity event
3. the activity code for the activity event
4. the location of the event

get_day_end (*day_start*, *start*, *dt*)

This function gets the day that an activity ends.

Parameters

- **day_start** (*numpy.ndarray*) – the day an activity starts
- **start** (*numpy.ndarray*) – the time an activity starts [hours]
- **dt** (*numpy.ndarray*) – the duration for an activity [hours]

Returns the day an activity ends

Return type numpy.ndarray

get_weekday_data (*df=None*)

This function pulls out data that only corresponds to the weekday data.

Parameters **df** (*pandas.core.frame.DataFrame*) – the activity-diary of interest. If df is None, then use the dataframe associated with the diary object

Returns the activity-diary of data that occur on weekdays

get_weekday_idx (*df=None*)

Get the indices of the data that occurs on weekdays. An activity is considered to be on the weekday if the activity **ends** on Monday - Friday.

Parameters **df** (*pandas.core.frame.DataFrame*) – the activity-diary of interest. If df is None, then use the dataframe associated with the diary object

Returns boolean indices of which activities end during the weekend

Return type numpy.ndarray

get_weekend_data (*df=None*)
This function pulls out data that only corresponds to the weekend data.

Parameters **df** (*pandas.core.frame.DataFrame*) – the activity-diary of interest. If df is None, the use the dataframe associated with the current diary object

Returns an activity-diary of data that occurs on weekends

get_weekend_idx (*df=None*)
Get the indices of the data that occurs on weekend. An activity is considered to be on the weekend if the activity **ends** on Saturday or Sunday.

Parameters **df** (*pandas.core.frame.DataFrame*) – the activity-diary of interest. If df is None, then use the dataframe associated with the diary object

Returns boolean indices of which activities end during the weekend

Return type numpy.ndarray

group_activity (*t*, *y*)
This function groups activities in chronological order.

Parameters

- **t** (*numpy.ndarray*) – the start time for activities
- **y** (*numpy.ndarray*) – the activity code that corresponds with the respective time

Returns a list of each unique group-lists. Each group-list contains a tuple for (time step, activity code)

group_activity_indices (*groups*)
This function returns the indices for each continuous activity in chronological order.

Note: The output is the time step number **not** the value of time

Parameters **groups** (*list*) – a list of tuples of (time step, activity code)

Returns

group_activity_key (*x*)
This is the key function used in groupby in order to group consecutive time-step-activity pairs. This is necessary for creating an activity diary.

Parameters **x** (*tuple*) – the data in the form of (index, (time step, activity code))

:return the key for sorting (, activity code) :rtype: tuple

is_weekend (*day*)
This function returns true if a day is in the weekend and false if it's in a weekday.

Parameters **day** (*numpy.ndarray*) – the day of the weekend

Returns boolean index of whether or not a day is in the weekend (True) or not (False)

Return type numpy.ndarray

same_day (*start*, *dt*)
This function returns true if the activity starts and ends on the same day.

Parameters

- **start** (*numpy.ndarray*) – the time an activity starts [hours]
- **dt** (*numpy.ndarray*) – the time an activity ends [hours]

Returns a boolean index of whether or not an activity started and ended on the same day

Return type numpy.ndarray

toString()
This expresses the diary as a string

Returns an expression of the diary as a string

Return type string

2.7 eat module

This module contains information about the activities associated with eating. This class is an `activity.Activity` that gives a `person.Person` the ability to eat and satisfy the need `hunger.Hunger`.

This module contains the following classes:

1. `eat.Eat` (general eating capabilities)
2. `eat.Eat_Breakfast` (eating breakfast)
3. `eat.Eat_Lunch` (eating lunch)
4. `eat.Eat_Dinner` (eating dinner)

Module author: Dr. Namdi Brandon

class eat.**Eat**
Bases: `activity.Activity`

This class has general capabilities that allow the person to eat in order to satisfy `hunger.Hunger`. This class acts as a parent class and is expected to inherited.

advertise(*p*)
This function handles advertising the score to an agent. This function returns 0.

Note: This function should be overloaded when inherited.

Parameters **p** (person.Person) – the person of interest

Returns the score (0)

Return type float

advertise_help(*p*, *dt*)
This function does some of the logistics needed for `advertise()`.

This function does the following:

1.sets the suggested recharge rate for hunger

2.calculates the score

Parameters

- **p** (person.Person) – the person who is being advertised to
- **dt** (*float*) – the duration of the activity

Returns the score

Return type float

advertise_interruption(*p*)

This function calculates the score of an activity advertisement when a person is going to interrupt an ongoing activity in order to do an eating activity.

This function does the following:

1. temporarily sets the satiation of hunger $n_{hunger}(t) = \eta_{interruption}$
2. calculate the score advertised for the potential eating activity that will interrupt a current activity
3. restores the the satiation for hunger to the original value

Parameters **p** (person.Person) – the person of interest

Return score the value of the advertisement

Return type float

end(*p*)

This function ends the eat activity.

Parameters **p** (person.Person) – the person whose activity is ending

Returns None

end_meal(*p*)

This function ends the eat activity by doing the following.

1. frees the person's use of the asset
2. sets the state to idle (`state.IDLE`)
3. sets the satiation of hunger
4. set the current meal for the next day
5. set any skipped meals to be on the next day
6. find the the next meal
7. sets the decay rate of hunger
8. update the scheduler so that hunger will trigger the schedule to stop at the next meal
9. set the next meal to the current meal

Parameters **p** (person.Person) – The person whose meal is ending.

Returns None

set_end_time(*p*)

This function returns the end time of eating (universal time).

Parameters **p** (person.Person) – the person of interest.

Return t_end the end time of eating [minutes, universal time]

Return type int

start(*p*)

This function starts the eating activity.

Parameters **p** (person.Person) – The person whose activity is starting.

Returns None

start_meal (*p*)

This function starts the eat activity by doing the following.

1.sets the person's state to busy (`state.BUSY`)

2.set the decay rate of hunger to 0

3.store the start time to the state

4.sets the end time

5.sets the hunger recharge rate

6.updates the asset's state and number of users

7.update the schedule for the hunger need to trigger when the eat activity is scheduled to end

Parameters **p** (person.Person) – the person who is starting the meal

Returns None

test_func (*p*)

class `eat.`**Eat_Breakfast**

Bases: *eat.Eat*

This class is used to handle the logistics for eating breakfast.

advertise (*p*)

This function calculates the score of an activities advertisement to a person.

Advertise to the agent if the following conditions are met

1.the current meal is breakfast

2.the agent's location is at home (`location.HOME`)

3.calculate the score

$$S = \begin{cases} 0 & n_{hunger}(t) > \lambda \\ W(n_{hunger}(t)) - W(n_{hunger}(t + \Delta t)) & n_{hunger}(t) \leq \lambda \end{cases}$$

Parameters **p** (person.Person) – the person of interest

Return score the advertised score of doing the eat breakfast activity

Return type float

end_meal (*p*)

This function handles the logistics for ending the eat activity by doing the following.

1.call `eat.end_meal()`

2.If planning to skip lunch, update the lunch event to be the next day

Parameters **p** (person.Person) – the person who's meal is ending

Returns

start_meal (*p*)

This function handles the logistics for starting the eat activity by doing the following

1. set the current meal to breakfast
2. call `eat.start_meal()`

Parameters **p** (person.Person) – the person who is starting the eat activity

Returns

class `eat.`**`Eat_Dinner`**

Bases: *`eat.Eat`*

This class is used to handle the logistics for eating dinner.

`advertise`(*p*)

This function calculates the score of an activities advertisement to a Person.

Advertise to the agent if the following conditions are met

1. the current meal is lunch
2. the agent's location is at home (`location.HOME`)
3. calculate the score

$$S = \begin{cases} 0 & n_{hunger}(t) > \lambda \\ W(n_{hunger}(t)) - W(n_{hunger}(t + \Delta t)) & n_{hunger}(t) \leq \lambda \end{cases}$$

If the threshold is not met, score is 0. The advertisements assume that the duration of the activity is the mean duration.

Parameters **p** (person.Person) – The person of interest

Return score the advertised score of doing the Eat activity

Return type float

`end_meal`(*p*)

This function goes through the logistics of ending the dinner meal by doing the following:

1. calls `end.end_meal()`
2. If breakfast will be skipped, update the lunch event to be 2 days from the current day

Parameters **p** (person.Person) – the person who is finishing the eating dinner event

Returns None

`start_meal`(*p*)

This function goes through the logistics of starting the dinner meal by doing the following:

1. set the current meal to dinner
2. call `eat.start_meal()`

Parameters **p** (person.Person) – the person who is starting the eat dinner event

Returns None

class `eat.`**`Eat_Lunch`**

Bases: *`eat.Eat`*

This class is used to handle the logistics for eating lunch.

advertise (*p*)

This function calculates the score of an activities advertisement to a person.

Advertise to the agent if the following conditions are met

1. the current meal is lunch
2. the agent's location is at home (`location.HOME`) or the agent's location is at the workplace (`location.OFF_SITE`)
3. calculate the score

$$S = \begin{cases} 0 & n_{hunger}(t) > \lambda \\ W(n_{hunger}(t)) - W(n_{hunger}(t + \Delta t)) & n_{hunger}(t) \leq \lambda \end{cases}$$

If the threshold is not met, score is 0. The advertisements assume that the duration of the activity is the mean duration.

Parameters **p** (person.Person) – The person of interest

Return score the advertised score of doing the Eat activity

Return type float

end_meal (*p*)

This function ends the eat activity by doing the following:

1. calls `eat.end_meal()`
2. **if dinner is to be skipped, update the dinner event by doing the following:**
    - **if the lunch is an interrupting activity**
        - set the time until the next lunch activity
        - update the schedule for the interruption to the next lunch activity
        - set the interruption state to False

Parameters **p** (person.Person) – The person whose meal is ending.

Returns None

start_meal (*p*)

This function handles the logistics for starting the eat activity by doing the following:

1. sets the current meal to lunch
2. call `eat.start_meal()`

Parameters **p** (person.Person) – the person starting the eat lunch event

Returns None

2.8 food module

This module contains information about the asset that allows for the eating activity.

This module contains the following class: `food.Food`.

Module author: Dr. Namdi Brandon.

class food.**Food**

Bases: *asset.Asset*

This class represents an asset that allows the agent to eat breakfast, eat lunch, and eat dinner.

Activities in this asset:

1. `eat_breakfast.Eat_Breakfast`
2. `eat_lunch.Eat_Lunch`
3. `eat_dinner.Eat_Dinner`

2.9 home module

This module governs the control of assets used in the simulation. Mainly, the home contains all of the assets used in the simulation for the current version of the code.

This module contains the following class: *home.Home*

Module author: Dr. Namdi Brandon.

class home.**Home**(*clock*)

Bases: `object`

Contains all of the physical characteristics of a home/ residence. Currently, the home contains all of the assets within the simulation.

Parameters **clock** (temporal.Temporal) – the time

Variables

- **assets** (*dict*) – contains a list of all of the assets available in the home.
- **category** (*int*) – the type of home
- **clock** (temporal.Temporal) – the time
- **id** (*int*) – a unique home identification number
- ***location*** (location.Location) – the location of the home
- **population** (*int*) – the number of people who reside in a home
- **revenue** (*float*) – the household revenue

advertise(*p*, *do_interruption=False*, *locale=None*)

This function handles all of the Activities' advertisements to a Person. This occurs by looping through each asset in the home and collecting a list of advertisements for each activity in each asset.

1. loop through each asset
2. **if the asset is busy *and* is in the same location of the person**
   - **for each activity in the given asset**
     (a) advertise for interrupting activities
     (b) advertise for non interrupting activities
     (c) collect the advertisements

Parameters

- **p** (person.Person) – a person to whom the assets are advertising

- **do_interruption** (*bool*) – a flag that indicates whether or not we should advertise for interruptions
- **locale** (*int*) – a local location identifier

Returns the advertisements (score, asset, activity, person) containing the various data for each advertisement: ("score", "asset", "activity", "person") coupling

Return type dict

initialize(*people*)
Initialize the assets in the home.

Parameters **people** (*list*) – a list of people who reside in the home

Returns None

print_category()
This function expresses the category variable as a string.

Returns string representation of the category

Return type str

reset()
This function resets the each asset in the home.

Returns None

set_population(*people*)
Set the population of the house.

Parameters **people** (*list*) – the list of people living in the home

Returns None

set_revenue(*people*)
Sets the revenue of the home by adding the revenue of each person in the home.

Parameters **people** (*list*) – the list of people living in the home

Returns None

toString()
This function expresses the Home object as a string

Return msg the string representation of the home object

Return type str

2.10 hunger module

This module contains information about governing the need Hunger.

This module contains the class hunger (*hunger.Hunger*).

Module author: Dr. Namdi Brandon

class hunger.**Hunger**(*clock*, *num_sample_points*)
Bases: *need.Need*

This class governs the behavior of the need Hunger need. When Hunger is unstatisfied, the agent feels compelled to eat a meal in order to satisfy the need. Mathematically speaking, Hunger is modeled as linearly-behaving need.

Parameters

- **clock** (temporal.Temporal) – the time
- **num_sample_points** (*int*) – the number of temporal nodes in the simulation

Variables

- **category** (*int*) – the category of the need
- **decay_rate** (*float*) – the decay rate of the Hunger need [need/minute]
- **recharge_rate** (*float*) – the recharge rate of the Hunger need [need/min]
- **suggested_recharge_rate** (*float*) – an approximate recharge rate used to calculate the end time of an event before rounding

decay (*status*)

This function decreases the satiation in Hunger by doing the following:

$$n(t+1) = n(t) + m_{decay}$$

> **Warning:** This function may be antiquated and **not used**

Parameters **status** (*int*) – indicates the current status of the person's state (not-used)

Returns None

decay_new (*dt*)

This function sets the default decrease in the Hunger need.

$$n(t+\Delta t) = n(t) + m_{decay}\,\Delta t$$

Parameters **dt** (*int*) – the duration of time [minutes] used to decay the need

Returns None

initialize (*p*)

This function initializes the the hunger need at the first step of the simulation. The function checks to see whether or not the current time implies that there should be an eating event. The hunger object is set to the respective state.

This function does the following exactly

1. initialize all of the meals
2. check to see if a meal should be occurring at the current time
3. **if no meals should be occurring**
   - figure out the next meal m
   - calculate the decay rate for hunger until the next meal
   - calculate the amount of time until the next meal Δt
   - set the current meal m
   - update the schedule for the hunger need to be the time the next meal starts
4. **if a meal should be occurring**
   - get the index of the meal that should be occurring m
   - set the current meal m
   - calculate the final time of the meal

- calculate the duration until the end of the next meal Δt
- set the recharge rate
- update the scheduler for the hunger need to be the time the current meal should end

5.initialize the start time for each meal

Parameters **p** (person.Person) – the person whose hunger need is being initialized

Returns None

is_meal_time (*t*, *the_meal*)
This checks whether or not it is time for a meal.

Parameters

- **t** (*int*) – time of day [minutes]
- **the_meal** (meal.Meal) – the respective meal to see whether the current time implies that an eating event should happen

Returns True if the current time is within the time to eat. False, otherwise

Return type bool

is_meal_time_all (*t*, *meals*)
This function checks every meal and sees whether or not the current time implies that there should be an eventing event for a respective meal

Parameters

- **t** (*int*) – The current time of day [minutes]
- **meals** (*list*) – A list of Meals

Returns A list of boolean flags indicating True or False, indicating whether or not an eating event should occur for the respective meal

Return type list

perceive (*future_clock*)
This gives the result if eat is done now until a later time corresponding to clock.

Parameters **future_clock** (temporal.Temporal) – a clock at a future time

Return out the perceived hunger need association level

Return type float

reset ()
This function resets the values in order for the need to be used in the next simulation :return:

set_decay_rate (*t_start*)
This function calculates the decay rate of hunger to the next meal.

Parameters

- **dt** (*int*) – the amount of time to the next meal [minutes]
- **t_start** (*int*) – the start time [in minutes] of the next meal

Returns None

set_decay_rate_new (*dt*)
This function calculates the decay rate of hunger to the next meal.

Parameters **dt** (*int*) – the amount of time to the next meal [minutes]

Returns None

set_recharge_rate(*dt*)
This function calculates the recharge rate of hunger due to eating the current meal.

Parameters **dt** (*int*) – the amount of time it takes to finish a meal [minutes]

Returns None

set_suggested_recharge_rate(*dt*)
This function sets the suggested recharge rate assuming a **linear function** behavior

The suggested recharge rate is based on the duration of the sleeping event and the threshold. The sleep duration is based on the biological data (no rounding).

Parameters **dt** (*int*) – The duration of time of the eating event [minutes]

Returns None

toString()

Represents the Hunger object as a string.

Return msg the string representation of the huger object

Return type str

2.11 income module

This is module contains code for governing the need to work/ be schooled.

This module contains the class *income.Income*.

Module author: Dr. Namdi Brandon

class income.**Income**(*clock*, *num_sample_points*)
Bases: *need.Need*

This class governs the need association dealing with work / school. Recall that income mathematically resembles a step function.

Parameters

- **clock** (temporal.Temporal) – the time
- **num_sample_points** (*int*) – the number of temporal node points in the simulation

decay(*p*)
This function decays the magnitude of the need. Income only decays after the job start time.

1. Find out if it is time to work
2. If it's time to work, set the satiation $n_{income} = \eta_{work}$

Parameters **p** (person.Person) – the person of interest

Returns None

initialize(*p*)
This function is used to initialize the agent's income need at the beginning of the simulation. This function initializes the Person to be at the workplace (`location.OFF_SITE`) if it is work time. This function does the following:

1. decay the income satiation
2. **if the person is supposed to be at work**
    - set the person to the workplace location
    - else, set the amount of time until the next work event
3. update the scheduler for the income need

Parameters **p** (person.Person) – the person of interest

Returns None

perceive(*clock*, *job*)
This gives the satiation of income **if** the income need is addressed now.

1. find out if the time associated with clock implies a work time for the person
2. **If it should be work time**
    - the perceived satiation is $\eta_{work} \leq \lambda$
    - else, the perceived satiation is 1.0

Parameters

- **clock** (temporal.Temporal) – the future time the activity the should be perceived to be done
- **job** (occupation.Occupation) – the job

Returns the satiation at the perceived time

Return type float

2.12 interrupt module

This module contains code for interrupting a current activity.

This module contains class *`interrupt.Interrupt`*.

Module author: Dr. Namdi Brandon

class `interrupt.`**`Interrupt`**
Bases: *`activity.Activity`*

This class allows for the current activity to be interrupted by another activity.

advertise(*p*, *str_need*, *act*)
This function calculates the score of an activities advertisement to a Person. This function does the the following:

1. temporarily sets the value of the Need that must be immediately addressed to a low level.
2. send an advertisement is is made from the potentially interrupting activity
3. calculate the score from the potentially interrupting activity

Parameters

- **p** (person.Person) – the person who is being advertised to
- **str_need** (*int*) – the id of the Need that needs to be addressed, which could potentially cause an interrupting event
- **act** (activity.Activity) – the activity of interest that could immediately interrupt a current activity

Returns the value of the advertisement

Return type float

start(*p*)

This handles the start of an activity.

Parameters **p** (person.Person) – the person of interest

Returns None

2.13 interruption module

This class gives an agent the ability to interrupt a current activity.

This module contains class *interruption.Interruption*.

Module author: Dr. Namdi Brandon

class interruption.**Interruption**(*clock*, *num_sample_points*)

Bases: *need.Need*

This class enables a Person to interrupt a current activity.

Interruption is a minor Need (need-association). That is, this need-association is NOT independent of other needs.

Parameters

- **clock** (temporal.Temporal) – The clock governing time in the simulation
- **num_sample_points** (*int*) – The number of time nodes in the simulation

Variables

- **category** (*int*) – the category of the interruption Need
- **activity_start** (*int*) – the category of the (interrupting) activity to start
- **activity_stop** (*int*) – the category of the (interrupted) activity to stop

decay(*p*)

This function sets the default decrease in the Interruption need

Parameters **p** (person.Person) – the person of interest

Returns None

get_time_to_next_work_lunch(*p*)

initialize(*p*)

Initializes the need during the first step

Parameters **p** (person.Person) – Initialize the state of the interruption need-association at the beginning of the simulation

Returns None

is_lunch_time (*time_of_day*, *meals*)
This function indicates whether it is lunch time or not. This is used in the interruption to stop the work activity and begin the eat lunch activity.

Parameters

- **time_of_day** (*int*) – the time of day [minutes]
- **meals** (*list*) – a list of the meals (*meal.Meal*) for the agents. Some of the entries in the list may be None.

Return is_lunch a flag indicating whether it is lunch time

perceive (*clock*)
This gives the result if sleep is done now until a later time corresponding to clock.

Parameters **clock** (temporal.Temporal) – a clock at a future time

Return out the perceived interruption magnitude

reset ()
This function resets the interruption need completely in order to re run the simulation. In this reset, the history is also reset.

Returns

reset_minor ()
This function resets the interruption need

Returns None

stop_work_to_eat (*p*)
This function checks to see if an interruption should occur to allow a Person to start the eating activity while doing the work activity

An agent may stop the work activity to eat lunch if the following conditions are met:

1. The agent is hungry
2. The current activity is work
3. It is lunch time

Parameters **p** (person.Person) – the person of interest

Returns None

2.14 location module

This module is responsible for containing information about the concept of location.

This module contains class *location.Location*.

Module author: Dr. Namdi Brandon

class location.**Location** (*geography=1*, *local=0*)
Bases: object

This class holds information relevant to the location of persons and assets.

Parameters

- **geography** (*int*) – the geographical location code
- **local** (*int*) – the local location code

Variables

- **geo** (*int*) – the geographical location code within the U.S (e.g. north, south, eats, or west)
- **local** (*int*) – the local location code (e.g. home, off site, etc)

print_geo()

Returns the geographical location in a string format

Returns the string representation of the geographical location

Return type str

print_local()

Returns the local location in a string format

Returns the string representation of the local location

Return type str

reset()

This function resets the location to the default value, (`location.HOME`).

Returns None

toString()

This function represents the Location object as a string.

Return msg the string representation of the Location object

Return type str

2.15 meal module

This module contains contains information about various meals that an agent would eat in.

This module contains code for class `meal.Meal`.

Module author: Dr. Namdi Brandon

class meal.**Meal**(*id=0*, *start_mean=390*, *start_std=10*, *start_trunc=1*, *dt_mean=15*, *dt_std=5*, *dt_trunc=1*)

Bases: `object`

This is class contains information about meals (breakfast, dinner, and lunch)

Variables

- **id** (*int*) – the meal type (breakfast, lunch, or dinner)
- **dt** (*int*) – the duration of a meal [minutes]
- **dt_mean** (*int*) – the mean duration of a meal [minutes]
- **dt_std** (*int*) – the standard deviation of meal duration [minutes]
- **dt_trunc** (*int*) – the number of standard deviation in the duration distribution
- **t_start** (*int*) – the start time of a meal [minutes, time of day]

- **t_start_univ** (*int*) – the start time of a meals [minutes, universal time]
- **start_mean** (*int*) – the mean start time of a meal [minutes, time of day]
- **start_std** (*int*) – the standard deviation of start time of a meal [minutes]
- **start_trunc** (*int*) – the number of standard deviation of in the start time distribution
- **f_start** – the start time distribution function
- **f_dt** – the duration distribution function
- **day** (*int*) – the day the meal should occur

initialize(*t_univ*)

At the beginning of the simulation, make sure that the meals are initialized to the proper times (`t_start_univ`) so that they relate to the simulation time (t_univ)

Parameters **t_univ** (*int*) – the simulation time [minutes, universal time]

Returns None

print_id()

This function returns a string representation of the meal id

Returns a string representation of the meal id

Return type str

set_meal(*id*, *start_mean*, *start_std*, *start_trunc*, *dt_mean*, *dt_std*, *dt_trunc*)

This function sets the values associated with the Meal object.

Parameters

- **id** (*int*) – the meal type (breakfast, lunch, or dinner)
- **start_mean** (*int*) – the mean start time of the meal [minutes, time of day]
- **start_std** (*int*) – the standard deviation of start time [minutes]
- **start_turnc** (*int*) – the number of standard deviations in the start time distribution
- **dt_mean** (*int*) – the mean duration of a meal [minutes]
- **dt_std** (*int*) – the standard deviation of meal duration [minutes]
- **dt_trunc** (*int*) – the number of standard deviations in the duration distribution

Returns None

toString()

This function returns a string representation of the Meal object.

Return msg a string representation of the Meal object

Return type str

update(*day*)

Given the day for the meal, update the meal. The following does the following:

1. Update the start time for the meal
2. Update the duration for the meal
3. Update the universal start time for the meal

Parameters **day** (*int*) – the day for the meal to occur

Returns None

update_day()
Update the day for the next meal, given the universal start time for the meal (`t_start_univ`).

Returns None

update_dt()
Sample the duration distribution to get a duration.

Returns None

update_start()
Sample the start time distribution to get a start time.

Returns None

update_start_univ(*day*)
Given the day for the next meal, update the universal start time for the meal.

Parameters **day** (*int*) – the day for the meal

Returns None

2.16 my_globals module

This module contains constants and functions that are used for general use.

This module contains information about the following constants:

1. Identifiers for activity codes
2. File names file paths for saving figures for the different demographics
3. File names file paths for saving figures for the different activities

Module author: Dr. Namdi Brandon

my_globals.**fill_out_data**(*t*, *y*)
This function takes an array of activity start times and activity codes from an activity diary and fills out the activity, minute-by-minute in between two adjacent activities.

Parameters

- **t** (*numpy.ndarray*) – the start times in an activity diary
- **y** (*numpy.ndarray*) – the activity codes in an activity diary

Returns

my_globals.**fill_out_time**(*t*)
This function takes an array of activity start times from an activity diary and fills out the time, minute-by-minute in between two adjacent activities

Example, if t = (0, 4, 7) (and $t_{final} = 10$) we get the following:

- (0, 1, 2, 3)
- (4, 5, 6)
- (7, 8, 9, 10)

Parameters **t** (*numpy.ndarray*) – the start times in the activity diary [minutes, universal time]

Returns None

my_globals.**from_periodic**(*t*, *do_hours=True*)
This function returns the time of day in a 24 hour format. It takes the time $t \in [-12, 12)$ and expresses it at time $x \in [0, 24)$ where 0 represents midnight. The same calculation can be done to represent the time in minutes

Parameters

- **t** (*float*) – the time in hours [-12, 12), or the respective minutes [-12 * 60, 12 * 60)
- **do_hours** (*bool*) – a flag to do the calculations in hours (if True)

Returns the time in [0, 24) or in minutes [0, 24 * 60)

my_globals.**get_ecdf**(*data*, *N=100*)
Given data, this function calculates the probability data from the empirical cumulative distribution function (ECDF).

Parameters

- **data** (*float*) – an array containing the relevant data to get the ECDF of
- **N** (*int*) – the amount of samples in calculating the ECDF results

Return y the ECDF

Return type float array

Return x the values sampled for the ECDF

Return type float array

my_globals.**group_time**(*t*)
This function takes data from an activity diary and groups that activity diary into , minute by minute arrays from start to end for each activity (start, start + 1, ... end-1, end)

Parameters **t** (*numpy.ndarray*) – the start times from an activity diary [minutes, universal time]

Returns the grouped start/end pairs for ech activitiy

Return type list

my_globals.**hours_to_minutes**(*t*)
This function takes a duration of time in hours and converts the time rounded to the nearest minutes.

Parameters **t** (*float*) – a duration of time [hours]

Returns the time in minutes

my_globals.**load**(*fname*)
This function loads data from a .pkl file.

Parameters **fname** (*str*) – the file name to be loaded from

Returns the data unpickled

my_globals.**sample_normal**(*std*, *dx*)
This function samples a normal distribution centered at zero assuming a max and minimum acceptable value [dx, -dx].

Parameters

- **std** (*float*) – the standard deviation
- **dx** (*float*) – the amount of total deviation from the mean allowd

Returns

my_globals.**save**(*x*, *fname*)
This function saves a python variable by pickling it.

Parameters

- **x** – the data to be saved
- **fname** (*str*) – the file name of the saved file. It must end with .pkl

my_globals.**save_zip**(*out_file*, *source_dir*)
This function compresses an entire directory as a zip file.

Parameters

- **out_file** (*str*) – the filename of the save zip file with out the .zip extension
- **source_dir** (*str*) – the directory to be compressed

Returns the name of the compressed directory

my_globals.**set_distribution**(*lower*, *upper*, *mu*, *std*)
This function sets the truncated normal probability distribution.

Parameters

- **lower** (*int*) – the lower bound in number of standard deviation from the mean
- **upper** (*int*) – the upper bound in number of standard deviation from the mean
- **mu** (*int*) – the mean
- **std** (*int*) – the standard deviation

Returns the function for the truncated normal distribution

my_globals.**set_distribution_dt**(*lower*, *upper*, *mu*, *std*, *x_min*)
This function set the truncated normal probability distribution subject to the fact that there is an assigned lowest value.

If the lowest value of the normal distribution is lower than the lowest allowed value, change the distribution so that the standard deviation allows the distribution to not be lower than the lowest allowed value.

Parameters

- **lower** (*int*) – the lower bound in number of standard deviation from the mean
- **upper** (*int*) – the upper bound in number of standard deviation from the mean
- **mu** (*int*) – the mean
- **std** (*int*) – the standard deviation
- **x_min** (*int*) – the lowest allowed value

Returns the function for the truncated normal distribution, the standard deviation of the distribution

Return type tuple

my_globals.**to_periodic**(*t*, *do_hours=True*)
This function returns the time of day in a periodic format. It takes the time $t \in [0, 24)$ and expresses it at time $x \in [-12, 12)$ where 0 represents midnight.

Parameters

- **t** (*float*) – the time in hours [0, 24)
- **do_hours** (*bool*) – a flag to do the calculations in hours (if True) or minutes if False

Returns the time in [-12, 12) or minutes [-12 * 60, 12 * 60)

Return type float

2.17 need module

This module contains information about governing the various needs that agents have in the simulation.

This module contains the class `need.Need`.

Module author: Dr. Namdi Brandon

class `need.`**`Need`**(*clock*, *num_sample_points*)

Bases: `object`

This class holds information about need-associations.

Parameters

- **`clock`** (temporal.Temporal) – the clock governing time in the simulation
- **`num_sample_points`** (*int*) – the number of time nodes in the simulation

Variables

- **`category`** (*int*) – the need- identifier
- **`clock`** (temporal.Temporal) – keeps track of the time
- **`history`** (*float*) – an array containing the magnitude level $[0, 1]$ of the need at all sample times.
- **`magnitude`** (*float*) – the magnitude of the need (the satiation)
- **`t0`** (*int*) – this keeps track of the last time the need was addressed
- **`threshold`** (*float*) – the threshold of the need

`decay`()

This calculates the amount of decay over a time step dt.

Note: This function is meant to be overridden.

Returns None

`initialize`()

This function initializes the state of the Need at the very beginning of simulation.

Note: This function is meant to be overridden.

Returns None

`print_category`()

This function represents the category as a string.

Returns the string representation of the category

Return type str

`reset`()

This function resets the values in order for the need to be used in the next simulation. This function does the following:

1.sets the satiation to 1.0

2.sets the history to zero

Returns None

toString()
This function represents the Need as a string.

Return msg The string representation of the Need object.

Return type str

under_threshold(*n*)
Compares the value of a Need to the threshold.

Parameters **n** (*float*) – the satiation

Returns True if the satiation is less than or equal to the threshold, False otherwise

Return type bool

weight(*n*)
This function calculates the weight function of a need.

Parameters **n** (*float*) – the satiation

Returns the weight due to the need

Return type float

2.18 occupation module

This module contains info needed for the occupation of a Person. In addition, this file also contains functions useful for the module itself.

This module contains class `occupation.Occupation`.

This module contains constants relevant to the occupational information:

- job identifiers
- job categories
- default start time information
- default end time information
- default commuting to work information
- default commuting from work information
- default summer vacation (from school) information

Module author: Dr. Namdi Brandon

class occupation.**Occupation**
Bases: object

This class contains information relevant to an occupation of a Person.

Variables

- **category** (*int*) – the category of the job
- **id** (*int*) – the identifier for the job
- **commute_to_work_dt_mean** (*int*) – the mean duration to commute to work [minutes]

- **commute_to_work_dt_std** (*int*) – the standard deviation to commute to work [minutes]
- **commute_to_work_dt** (*int*) – the duration to commute to work [minutes]
- **commute_to_work_dt_trunc** (*int*) – the number of standard deviation in the commute to work duration distribution
- **commute_to_work_start** (*int*) – the start time for the commute to work activity [minutes]
- **dt_commute** (*int*) – the duration of the commute [minutes]
- **dt** (*int*) – the duration of the work activity [minutes]
- **commute_from_work_dt_mean** (*int*) – the mean duration to commute from work [minutes]
- **commute_from_work_dt_std** (*int*) – the standard deviation to commute from work [minutes]
- **commute_from_work_dt** (*int*) – the duration to commute from work [minutes]
- **commute_from_work_dt_trunc** (*int*) – the number of standard deviations in the commute from work duration distribution
- **t_start_mean** (*int*) – the mean start time for the job [minutes, time of day]
- **t_start_std** (*int*) – the standard deviation of the start time for the job
- **t_start** (*int*) – the start time for the job [minutes, time of day]
- **t_start_univ** (*int*) – the start time for the job [minutes, universal time]
- **work_start_trunc** (*int*) – the number of standard deviations in the work start time distribution
- **day_start** (*int*) – the day the work activity start [minutes]
- **t_end_mean** (*int*) – the mean end time for the job [minutes, time of day]
- **t_end_std** (*int*) – the standard deviation of the end time for the job
- **t_end** (*int*) – the end time for the job [minutes, time of day]
- **t_end_univ** (*int*) – the end time for the job [minutes, universal time]
- **work_end_trunc** (*int*) – the number of standard deviations in the work end time distribution
- **is_employed** (*bool*) – this is a flag saying whether this is a job or not
- **is_same_day** (*bool*) – This is a flag to see whether the start time and end time of a job are on the same day. If so, True. Else, False. If a person has `NO_JOB`, the flag is set to True
- **'location'** (location.Location) – the location of the Occupation
- **wage** (*float*) – the yearly wage for that job [U.S. dollars]
- **work_days** (*list*) – a list of ints, giving the days the job starts
- **f_commute_to_work_dt** – the commute to work duration distribution
- **f_commute_from_work_dt** – the commute from work duration distribution
- **f_work_start** – the work start time distribution

- **f_work_end** – the work end time distribution

is_summer_vacation(*week_of_year*)
This function returns True if the agent should not go to school due to summer vacation. False, otherwise.

Parameters **week_of_year** (*int*) – the week of the year

Returns

print_category()
This function represents the Occupation category as a string

Returns the string representation of a Occupation category

Return type str

print_id()
This function writes the Occupation id as a string

Returns a string representation of the job ID

Return type str

set_commute_distribution()
This function sets the following:

- commute to work duration distribution
- commute from work duration distribution.

Returns None

set_is_job()
This function checks to see if the current job is actually a job (eg. that it is not `NO_JOB`).

Sets self.is_job to True if the Occupation is `NO_JOB`, returns False otherwise

Returns None

set_is_same_day()
This function sets a flag indicating whether or not a job starts and ends on the same day. The function sets `is_same_day` to True if the Occupation start time and end time are within the same day. False, otherwise.

Returns None

set_job_params(*id_job*, *start_mean*, *start_std*, *end_mean*, *end_std*, *commute_to_work_dt_mean*, *commute_to_work_dt_std*, *commute_from_work_dt_mean*, *commute_from_work_dt_std*)
This function sets the Occupation parameters.

Parameters

- **id_job** (*int*) – the job identifier
- **start_mean** (*int*) – the mean start time for the occupation
- **start_std** (*int*) – the standard deviation of the start time for the occupation
- **end_mean** (*int*) – the mean end time for the occupation
- **end_std** (*int*) – the standard deviation for the end time
- **commute_to_work_dt_mean** (*int*) – the mean commute to work duration
- **commute_to_work_dt_std** (*int*) – the standard deviation of the commute to work duration

- **commute_from_work_dt_mean** (*int*) – the mean commute from work duration
- **commute_from_work_dt_std** (*int*) – the standard deviation to commute from work duration

Returns None

set_job_preset()

Sets Occupation to one of the following preset jobs:

- `NO_JOB`
- `STANDARD_JOB`
- `STUDENT`

Returns None

set_no_job()

Set the Occupation to having no job.

Parameters **job** (occupation.Occupation) – the job of which to set the attributes

Returns None

set_standard_job()

This function sets the Occupation to the standard job.

- has fixed shift of 9:00 - 17:00
- Monday through Friday
- wage of $40,000
- 30 minute commute to work
- 60 minute commute from work

Parameters **job** (occupation.Occupation) – the job of which to set the attributes

Returns None

set_student()

This function sets the Occupation to the standard job.

- Fixed shift of 8:00 - 15:00
- Monday through Friday
- wage of $0
- 30 minute commute to school
- 60 minute commute from school

Parameters **job** (occupation.Occupation) – the job of which to set the attributes

Returns None

set_work_distribution()

This function sets the following distributions for work:

- work start time distribution
- work end time distribution

Returns None

toString()
Represents the Occupation object as a string

Return msg The representation of the Occupation object as a string

Return type str

update_commute_from_work_dt()
Pull a commute from work duration from the respective distribution.

Returns None

update_commute_to_work_dt()
Pull a commute to work duration from the respective distribution. Also, update the commute to work start time place holder.

Returns None

update_commute_to_work_start()
Update the commute to work start time.

Returns None

update_work_dt()
Update the work duration

Returns None

update_work_end()
Update the work end time.

Returns None

update_work_start()
Update the work start time.

Returns None

occupation.**is_work_time**(*clock*, *job*, *is_commute_to_work=False*)
Given a clock and a job, this function says whether the clock's time corresponds to a time to be at work **or** a time to commute to work.

If $\Delta t > 0$, it indicates when it's time to commute to work.

Parameters

- **clock** (temporal.Temporal) – the time
- **job** (occupation.Occupation) – the job to inquiry
- **is_commute_to_work** (*bool*) – a flag indicating whether we are interested in calculating if it is time to commute to work

Returns a flag indicating if it is / is not work time (or commute time if is_commute_to_work is True)

Return type bool

occupation.**is_work_time_help**(*clock*, *job*)
Given a clock and a job, this function says whether the clock's time corresponds to a time at work.

Parameters

- **clock** (temporal.Temporal) – the time

- **job** (occupation.Occupation) – the job to inquiry

Returns is_work_time: a flag indicating if the time (clock) corresponds to a work time

Return type bool

occupation.**set_grave_shift**(*job*)

This function sets the Occupation to a grave shift.

- shift job from 22:00 to 6:00
- Monday through Friday
- 30 minute commute to work
- 60 minute commute from work
- wage of $40,0000.

Parameters **job** (occupation.Occupation) – the job of which to set the attributes

Returns None

occupation.**set_no_job**(*job*)

Set the Occupation to having no job.

Parameters **job** (occupation.Occupation) – the job of which to set the attributes

Returns None

occupation.**set_standard_job**(*job*)

This function sets the Occupation to the standard job.

- fixed shift of 9:00 - 17:00
- Monday through Friday
- wage $40,000
- 30 minute commute to work
- 60 minute commute from work

Parameters **job** (occupation.Occupation) – the job of which to set the attributes

Returns None

occupation.**set_student**(*job*)

This function sets a job to the preset values of student occupation.

- fixed shift of 08:00 - 15:00
- Monday through Friday
- wage of $0
- 30 minute commute to school
- 60 minute commute from school

Parameters **job** (occupation.Occupation) – the job to set

Returns None

2.19 params module

The purpose of this module is to assign parameters necessary to run the Agent-Based Model of Human Activity Patterns (ABMHAP) initialized with data from the Consolidated Human Activity Database (CHAD). This module also have constants used in default runs.

This module contains class `params.Params`.

Module author: Dr. Namdi Brandon

class `params.`**`Params`**(*dt=1, num_people=1, num_days=1, num_hours=0, num_min=0, t_start=1865, gender=None, sleep_start_mean=None, sleep_start_std=None, sleep_end_mean=None, sleep_end_std=None, job_id=None, do_alarm=None, dt_alarm=None, bf_start_mean=None, bf_start_std=None, bf_start_trunc=None, bf_dt_mean=None, bf_dt_std=None, bf_dt_trunc=None, lunch_start_mean=None, lunch_start_std=None, lunch_start_trunc=None, lunch_dt_mean=None, lunch_dt_std=None, lunch_dt_trunc=None, dinner_start_mean=None, dinner_start_std=None, dinner_start_trunc=None, dinner_dt_mean=None, dinner_dt_std=None, dinner_dt_trunc=None, work_start_mean=None, work_start_std=None, work_end_mean=None, work_end_std=None, commute_to_work_dt_mean=None, commute_to_work_dt_std=None, commute_from_work_dt_mean=None, commute_from_work_dt_std=None*)

Bases: `object`

This class contains the parameters that are needed to parametrize a household.

Note: Some of the class attributes are **not** really used and need to be phased out in future versions of the model. Some of these attribtues are:

- `dt`
- `do_alarm`
- `dt_alarm`

Parameters

- **`dt`** (*int*) – the step size [in minutes] in the simulation
- **`num_people`** (*int*) – the number of people in the household
- **`num_days`** (*int*) – the number of days in the simulation
- **`num_hours`** (*int*) – the number of additional hours in the simulation
- **`num_min`** (*int*) – the number of additional minutes in the simulation
- **`t_start`** (*int*) – the start time [in minutes] in the simulation
- **`gender`** (*list*) – the gender of each person in the household
- **`sleep_start_mean`** (*list*) – the mean sleep start time [in minutes, time of day] for each person in the household
- **`sleep_start_std`** (*list*) – the standard deviation of sleep start time [in minutes] for each person in the household
- **`sleep_end_mean`** (*list*) – the mean sleep end time [in minutes, time of day] for each person in the household
- **`sleep_end_std`** (*list*) – the standard deviation of the sleep end time [in minutes] for each person in the household

- **job_id** (*list*) – the occupation identifier for each person in the household
- **do_alarm** (*list*) – a flag indicating whether or not a person uses an alarm for each person in the household
- **dt_alarm** (*list*) – the duration of time [in minutes] before an alarm goes off before its respective event
- **numpy.ndarray** – bf_start_mean: the mean breakfast start time for each person in the household [minutes, time of day]
- **bf_start_std** (*numpy.ndarray*) – the standard deviation for breakfast start time for each person in the household [minutes]
- **bf_start_trunc** (*numpy.ndarray*) – the number of standard deviations used in the breakfast start time distribution for each person
- **bf_dt_mean** (*numpy.ndarray*) – the mean breakfast duration for each person in the household [minutes]
- **bf_dt_std** (*numpy.ndarray*) – the standard deviation for breakfast duration for each person in the household [minutes]
- **bf_dt_trunc** (*numpy.ndarray*) – the number of standard deviations used in the breakfast duration distribution for each person
- **lunch_dt_mean** (*numpy.ndarray*) – the mean lunch duration for each person in the household [minutes]
- **lunch_dt_std** (*numpy.ndarray*) – the standard deviation for lunch duration for each person in the household [minutes]
- **lunch_dt_trunc** (*numpy.ndarray*) – the number of standard deviations used in the lunch duration distribution for each person
- **lunch_start_mean** (*numpy.ndarray*) – the mean lunch start time for each person in the household [minutes, time of day]
- **lunch_start_std** (*numpy.ndarray*) – the standard deviation for lunch start time for each person in the household [minutes]
- **lunch_start_trunc** (*numpy.ndarray*) – the number of standard deviations used in the lunch start time distribution for each person
- **dinner_start_mean** (*numpy.ndarray*) – the mean dinner start time for each person in the household [minutes, time of day]
- **dinner_start_std** (*numpy.ndarray*) – the standard deviation for dinner start time for each person in the household [minutes]
- **dinner_start_trunc** (*numpy.ndarray*) – the number of standard deviations used in the dinner start time distribution for each person
- **dinner_dt_mean** (*numpy.ndarray*) – the mean dinner duration for each person in the household [minutes]
- **dinner_dt_std** (*numpy.ndarray*) – the standard deviation for dinner duration for each person in the household [minutes]
- **dinner_dt_trunc** (*numpy.ndarray*) – the number of standard deviations used in the dinner duration distribution for each person
- **work_start_mean** (*numpy.ndarray*) – the mean work start time for each person in the household [minutes, time of day]

- **work_start_std** (*numpy.ndarray*) – the standard deviation of work start time for each person in the household [minutes]
- **work_end_mean** (*numpy.ndarray*) – the work end time for each person in the household [minutes, time of day]
- **work_end_std** (*numpy.ndarray*) – the work standard deviation for each person in the household [minutes, time of day]
- **commute_to_work_dt_mean** (*numpy.ndarray*) – the mean duration for commuting to work [minutes] for each person in the household
- **commute_to_work_dt_std** (*numpy.ndarray*) – the standard deviation for commuting to work [minutes] for each person in the household
- **commute_from_work_dt_mean** (*numpy.ndarray*) – the mean duration for commuting from work [minutes] for each person in the household
- **commute_from_work_dt_std** (*numpy.ndarray*) – the standard deviation for commuting from work [minutes] for each person in the household

Variables

- **dt** (*int*) – the step size [in minutes] in the simulation
- **num_people** (*int*) – the number of people in the household
- **num_days** (*int*) – the number of days in the simulation
- **num_hours** (*int*) – the number of additional hours in the simulation
- **num_min** (*int*) – the number of additional minutes in the simulation
- **t_start** (*int*) – the start time [in minutes] in the simulation
- **gender** (*list*) – the gender of each person in the household
- **sleep_start_mean** (*list*) – the mean sleep start time [in minutes, time of day] for each person in the household
- **sleep_start_std** (*list*) – the standard deviation of sleep start time [in minutes] for each person in the household
- **sleep_end_mean** (*list*) – the mean sleep end time [in minutes, time of day] for each person in the household
- **sleep_end_std** (*list*) – the standard deviation of the sleep end time [in minutes] for each person in the household
- **job_id** (*list*) – the occupation identifier for each person in the household
- **do_alarm** (*list*) – a flag indicating whether or not a person uses an alarm for each person in the household
- **dt_alarm** (*list*) – the duration of time [in minutes] before an alarm goes off before its respective event
- **breakfasts** (*list*) – the breakfast meal objects for each person in the household
- **lunches** (*list*) – the lunch meal objects for each person in the household
- **dinners** (*list*) – the dinner meal objects for each person in the household
- **work_start_mean** (*numpy.ndarray*) – the mean work start time for each person in the household [minutes, time of day]

- **work_start_std** (*numpy.ndarray*) – the standard deviation of work start time for each person in the household [minutes]
- **work_end_mean** (*numpy.ndarray*) – the work end time for each person in the household [minutes, time of day]
- **work_end_std** (*numpy.ndarray*) – the work standard deviation for each person in the household [minutes, time of day]
- **commute_to_work_dt_mean** (*numpy.ndarray*) – the mean duration for commuting to work [minutes] for each person in the household
- **commute_to_work_dt_std** (*numpy.ndarray*) – the standard deviation for commuting to work [minutes] for each person in the household
- **commute_from_work_dt_mean** (*numpy.ndarray*) – the mean duration for commuting from work [minutes] for each person in the household
- **commute_from_work_dt_std** (*numpy.ndarray*) – the standard deviation for commuting from work [minutes] for each person in the household

init_help (*val*, *default_val*)

This function assigns a default value to an attribute in case it was not assigned already. This is, function is particularly useful if the value to be assigned is an array depending on `num_people`

More specifically,

- If val is not None, return val
- If val is None, return the default value (default_val)

Parameters

- **val** – the value to be assigned
- **default_val** – the default value to assign in case val is None

Returns the non-None value

init_meal (*m_id*, *start_mean=None*, *start_std=None*, *start_trunc=None*, *dt_mean=None*, *dt_std=None*, *dt_trunc=None*)

This function returns the data for each person in the household for the respective meal given by "m_id"

- if specific parameters have been assigned, create meals with the respective parameters
- if specific parameters have not been assigned, create meals with the default meal parameters for each meal

Parameters

- **m_id** (*int*) – the identifier of meal type
- **start_mean** (*numpy.ndarray*) – the mean start time for the meal for each person in the household
- **start_std** (*numpy.ndarray*) – the standard deviation of start time for the meal for each person in the household
- **start_trunc** (*numpy.ndarray*) – the amount of standard deviations allowed before truncating the start time distribution for each person in the household
- **dt_mean** (*numpy.ndarray*) – the mean duration for the meal for each person in the household

- **dt_std** (*numpy.ndarray*) – the standard deviation for the meal for each person in the household
- **dt_trunc** (*numpy.ndarray*) – the amount of standard deviations allowed before truncating the duration distribution for each person in the household

Returns the meals for each person in the household

Return type list

init_meal_old (*id*, *start_mean=None*, *start_std=None*, *dt_mean=None*, *dt_std=None*)

This function returns the data for each person in the household for the respective meal given by “id”

> **Warning:** This function may be **not** used because it is antiquated.

Parameters

- **id** (*int*) – the id of meal type
- **start_mean** (*numpy.ndarray*) – the mean start time for the meal for each person in the household
- **dt_mean** (*numpy.ndarray*) – the mean duration for the meal for each person in the household
- **dt_std** (*numpy.ndarray*) – the mean standard deviation for the meal for each person in the household

Returns the meals for each person in the household

Return type list

set_num_steps ()

This function calculates and sets the number of time steps the ABM will run.

Return type None

tester ()

> **Warning:** This function is just for testing. It checks to see whether the expected dinner time is before the expected end time for work.

Returns

toString ()

This function represents the `params.Params` object as a string. For now, it prints the tuple (start time, duration, end time) in hours[0, 24] for the following activities:

1.eat breakfast

2.commute to work

3.work

4.eat lunch

5.commute from work

6.eat dinner

7.sleep

in order of start time. The commute activities only have duration information.

Returns the parameter information

2.20 person module

This module has code that governs information about the agent.

This module contains information about class `person.Person`.

Module author: Dr. Namdi Brandon

class `person.`**`Person`**(*house*, *clock*, *schedule*)

Bases: `object`

This class contains all of the information relevant for a Person.

A person is parametrized by the following

- a place of residence
- a biology
- social behavior
- a location
- a history of activities and states
- **Needs**
  1. Hunger
  2. Rest
  3. Income
  4. Travel
  5. Interruption

Parameters

- **house** (home.Home) – the Home object the Person resides in. (will need to remove this)
- **clock** (temporal.Temporal) – the time
- **schedule** (scheduler.Scheduler) – the schedule

Variables

- **'bio'** (bio.Bio) – the biological characteristics
- **clock** (temporal.Temporal) – keeps track of the current time. It is linked to the Universe clock
- **hist_state** (*numpy.ndarray*) – the state history [int] for each time step
- **hist_activity** (*numpy.ndarray*) – the activity history [int] for each time step
- **'home'** (home.Home) – this contains the place where the Person resides
- **id** (*int*) – unique person identifier
- **'income'** (income.Income) – the need that concerns itself with working/school
- **'interruption'** (interruption.Interruption) – the need that concerns itself with interrupting an ongoing activity
- **'location'** (location.Location) – the location data of a Person
- **needs** (*dict*) – a dictionary of all of the needs

- **'rest'** (rest.Rest) – the need that concerns itself with sleeping
- **socio** (social.Social) – the social characteristics of a Person
- **'state'** (state.State) – information about a Person's state
- **'travel'** (travel.Travel) – the need that concerns itself with moving from one area to another
- **hist_state** – the state of the person at each time step
- **hist_activity** – the activity code of the person at each time step
- **hist_local** (*numpy.ndarray*) – the location code of the person at each time step
- **H** (*numpy.ndarray*) – the satiation level for each need at each time step
- **need_vector** (*numpy.ndarray*) – the satiation level for each need at a given time step

print_basic_info()

This function expresses basic information about the Person as a string.

This prints the following:

- person identifier
- home identifier
- age
- gender

Returns basic information about the Person

Return type str

reset()

This function rests the person at the beginning of a simulation by doing the following:

1. reset the history
2. reset the state
3. reset the location
4. reset the needs

Note: the clock needs to be set to the beginning of simulation

Returns None

reset_history()

This function resets the variables:

1. history of the state
2. history of the activity
3. history of the location

Returns None

reset_needs()

This function resets the needs.

Returns None

toString()

This function represents the Person object as a string.

Returns information about the Person

Return type str

update_history()

This function updates the history of the following values with their current values

- state history
- location history
- activity history
- need (satiation) history

Returns

update_history_activity()

This function updates the activity history with the current values.

Returns None

update_history_needs()

This function updates the needs (satiation) history with the current values.

Returns None

2.21 rest module

This file contains information about the need dealing with resting.

This module contains class `rest.Rest`.

Module author: Dr. Namdi Brandon

class rest.**Rest**(*clock*, *num_sample_points*)

Bases: `need.Need`

This class contains relevant information about the rest need.

Parameters

- **clock** (temporal.Temporal) – this keeps track of the current time. It is linked to the Universe clock.
- **num_sample_points** (*int*) – the number of temporal nodes in the simulation

decay(*status*)

> **Warning:** This function is old and antiquated.

This function decays the Rest magnitude

Rest only decays if the person is **not** asleep. The decay in sleep

$$\delta = m_{decay}\Delta t$$
$$n(t + \Delta t) = n(t) + \delta$$

where

- m_{decay} is the decay rate
- Δt is the duration of time in 1 time step of simulation [minutes]
- δ is the amount of decay of rest
- $n(t)$ is the satiation at time t

Parameters **status** (*int*) – the current state of a person

Returns None

decay_new (*status*, *dt*)

This function decays the Rest magnitude

Rest only decays if the person is NOT asleep. The decay in sleep

$$\delta = m_{decay}\Delta t$$
$$n(t+\Delta t) = n(t) + \delta$$

where

- m_{decay} is the decay rate
- Δt is the duration of time in 1 time step of simulation [minutes]
- δ is the amount of decay of Rest
- $n(t)$ is the satiation at time t

Parameters

- **status** (*int*) – the current state of a person
- **dt** (*int*) – the duration of time [minutes] used to decay the need

Returns None

initialize (*p*)

The purpose of this code is to help initialize the Rest need and whatever activity that goes with it, depending on any time the simulation begins

Note: This code is a work in progress

1. update the sleep start and end time
2. find out if the person should be asleep
3. **if the Person is asleep,**
   - sets the appropriate duration of sleep left to do
   - sets the rest magnitude to threshold
   - sets the rest recharge rate
   - sets the schedule to trigger when when the person is scheduled to wake up
4. **if the Person is not asleep,**
   - sets the decay rate
   - set the magnitude

- sets the schedule to trigger when when the person is scheduled to start sleeping

5.update the schedule for the rest need

Parameters **p** (person.Person) – the person of interest

Returns None

is_workday(*p*)

This function indicates whether or not the sleep event resembles that from a person sleeping for a workday.

Parameters **socio** (social.Social) – the social characteristics of the person of interest

Returns True, if the sleep event resembles a workday. False, otherwise.

perceive(*future_clock*)

This functions gives the updated rest magnitude if sleep is done from now until a later time corresponding to clock.

$$\delta = m_{suggested} \Delta t$$

where

- $m_{suggested}$ is the suggested recharge rate
- Δt **is the duration of time from now until the future time** given by future_clock

Parameters **future_clock** (temporal.Temporal) – a clock corresponding to a future time

Returns the perceived rest level

Return type float

reset()

This function resets the values in order for the need to be used in the next simulation

Returns None

set_decay_rate(*dt*)

This function sets the decay rate.

The decay rate (m_{decay}) is assumed to be the slope of a linear function

where

- Δt is the duration of time expected to be awake
- λ is the rest threshold

$$m_{decay} = \frac{-1 + \lambda}{\Delta t}$$

Parameters **dt** (*int*) – the duration of sleep [minutes]

Returns None

set_recharge_rate(*dt*)

This function sets the recharge rate.

The recharge rate ($m_{recharge}$) is assumed to be the slope of a linear function

where

- Δt is the duration of sleep

- λ is the rest need threshold

$$m_{recharge} = \frac{1 - \lambda}{\Delta t}$$

Parameters **dt** (*int*) – the duration of sleep after rounding [minutes]

Returns None

set_suggested_recharge_rate (*dt*)

This function sets the "suggested" recharge rate. That is, the rate of recharge assuming exact arithmetic (there is no rounding in time, say to the nearest minute)

where

- Δt is the duration of sleep
- λ is the rest need threshold

$$m_{suggested} = \frac{1 - \lambda}{\Delta t}$$

Parameters **dt** (*int*) – the duration of sleep [minutes]

Returns None

should_be_asleep (*t_start*, *t_end*)

This function finds out if the person should be asleep for the initialization of the ABM module

Parameters

- **t_start** (*int*) – start time of sleep [minutes, time of day]
- **t_end** (*int*) – end time of sleep [minutes, time of day]

Returns a flag indicating whether a Person should be asleep (if True) or awake (if False)

Return type bool

toString ()

Represent the Rest object as a string

Returns the representation of the Rest object

Return type str

2.22 scheduler module

This module contains code that is is responsible for controlling the scheduler for the simulation. Note that the simulation does **not** run continuously in from one adjacent time step to the next. Instead the simulation jumps forward in time (i.e. move across multiple time steps in time), stopping only at time steps in which an action could occur. The ability to jump forward in time is controlled by the scheduler.

The scheduler will trigger the simulation to stop skipping time steps for the following reasons:

1. an activity should start
2. an activity should end
3. a need is under threshold

This module contains class `scheduler.Scheduler`.

Module author: Dr. Namdi Brandon

class `scheduler.`**`Scheduler`**(*clock*, *num_people*)

Bases: `object`

This class contains the code for the scheduler. The scheduler is in charge of jumping forward in time and stopping at only potentially relevant time steps. The scheduler keeps track of the needs for every person in in the household and stops at time steps where any person should have an action / need that needs to be addressed.

Parameters

- **`clock`** (temporal.Temporal) – the time
- **`num_people`** (*int*) – the number of people in the household

Variables

- **`clock`** (temporal.Temporal) – the time
- **`A`** (*numpy.ndarray*) – the schedule matrix of dimension (number of people x number of needs). This matrix contains the times [minutes, universal time] that the simulation should not skip over
- **`dt`** (*int*) – the duration of time between events
- **`t_old`** (*int*) – the time [minutes, universal time] of the prior event

`get_next_event_time`()

This function searches the schedule matrix and finds the next time that that model should handle.

Note: This function is only capable of handling **single-occupancy** households.

Returns the next time [minutes, time of day] that the model should address

Return type int

`toString`()

This function presents the Scheduler object as a string.

Returns a string representation of the object

`update`(*id_person*, *id_need*, *dt*)

This function updates the schedule matrix for a given person and need with the duration for the next event, for the respective person-need combination.

Parameters

- **`id_person`** (*int*) – the person identifier
- **`id_need`** (*int*) – the need identifier
- **`dt`** (*int*) – the duration to the next event

Returns None

2.23 sleep module

This module contains information about the activity dealing with sleeping. This class is *`activity.Activity`* that gives a *`person.Person`* the ability to eat and satisfy the need *`rest.Rest`*.

This file contains class *`sleep.Sleep`*.

Module author: Dr. Namdi Brandon

class `sleep.Sleep`

Bases: *activity.Activity*

This class is responsible for the act of sleeping, which satisfies the need *rest.Rest*.

advertise(*p*)

This function calculates the score of an activity advertisement to a Person

Parameters **p** (person.Person) – the person being advertised to

Returns the value of the advertisement

Return type float

end(*p*)

This handles the end of the sleep activity.

Parameters **p** (person.Person) – the person of interest

Returns None

end_sleep(*p*)

This function addresses logistics with a person waking up from sleep

1. free the asset from use
2. set the state of the person to idle (`state.IDLE`)
3. update the satiation
4. update the start time and end time
5. set the decay rate
6. update the schedule for the rest need

Parameters **p** (person.Person) – the person of interest

Returns None

is_workday(*p*)

This function indicates whether or not the sleep event resembles that from a person sleeping for a workday.

Parameters **p** (person.Person) – the person of interest

Returns True, if the sleep event resembles a workday. False, otherwise.

set_end_time(*p*)

This function returns the end time of sleeping

The end time t_{end} is set as follows

$$\begin{cases} \Delta t &= \frac{1-n(t)}{m_{suggested}} \\ t_{end} &= t + \Delta t \end{cases}$$

where

- Δt is the duration of sleep
- $m_{suggested}$ is the suggested recharge rate
- $n(t)$ is the magnitude of sleep at time t

Parameters **p** (person.Person) – the person of interest

Return t_end the end time of the sleep event [minutes, universal time]

Return type int

start (*p*)
This handles the start of the sleep activity.

Parameters **p** (person.Person) – the person of interest

Returns None

start_sleep (*p*)
This handles what happens when a Person goes to sleep.

1. The asset's status is updated.
2. The Person's state is set to the sleep state (`state.SLEEP`)
3. The end time is calculated
4. The recharge rate is set (according to whether or not it is a workday / non-workday)

Parameters **p** (person.Person) – the person of interest

Returns None

toString ()
This function represents the Sleep object as a string

Return msg the representation of the Sleep object

Return type str

2.24 social module

This module contains code that governs the social behavior/ characteristics relevant to a Person.

This module contains class *`social.Social`*.

Module author: Dr. Namdi Brandon

class `social.`**Social** (*age*, *num_meals=3*)
Bases: `object`

This class contains all of the relevant information governing the Person's social behavior.

Note: The current version of the ABM does not have any "alarm" functionality / capability. The remnants of any code that governs the use of an alarm will be removed in future updates.

Parameters

- **age** (*int*) – the age of the Person [years]
- **num_meals** (*int*) – the number of meals per day

Variables

- **is_child** (*bool*) – this flag is True if the Person is a child, False otherwise
- **job** (occupation.Occupation) – the information pertaining the the job
- **num_meals** (*int*) – the number of meals per day a person will eat
- **meals** (*list*) – a list of the meals that a person eats (*`meal.Meal`*)
- **current_meal** (meal.Meal) – the meal that is currently being eaten **or** if the Person is not eating a meal, it is the upcoming meal

- **next_meal** (meal.Meal) – the meal that is after the meal indicated by `current_meal`
- **uses_alarm** (*bool*) – indicates whether or not a person uses an alarm to wake up
- **is_alarm_set** (*bool*) – indicates whether or not an alarm is set for the current day
- **t_alarm** (*int*) – the time an alarm is supposed to go off [minutes, time of day]

duration_to_next_commute_event (*clock*)

This function is called in in order to calculate the amount of time until the next commute event by doing the following.

1. If the agent is unemployed, return infinity
2. If the time indicates that the agent should be currently working, set the duration to be the length of time remaining at work
3. If the time indicates that the agent should be currently commuting to work, set the duration to be the duration until the commute to work should start
4. If the time indicates that the agent should be currently commuting from work, set the duration to be the amount of time until the commute from work should end
5. Else, calculate the amount of time until the next commute to work event

Note: The only reason this code is place here is because the work activity and the commute activity use it.

Parameters **clock** (temporal.Temporal) – the current time

Returns the duration in time [minutes] until the next commute event

Return type int

duration_to_next_meal (*t_univ*)

This function calculates the amount of time until the next meal.

Parameters **t_univ** (*int*) – the current time [minutes, universal time]

Returns the duration to the next meal [minutes]

Return type int

Returns the scheduled next meal

Return type *meal.Meal*

duration_to_work_event (*clock*)

This function is called in in order to calculate the amount of time until the next work event.

1. If the person is employed, the duration to the next meal is set to infinity
2. **If the current time is a workday before the time work starts,**
   - set the duration to the amount of time until the start of work
3. **Else,**
   - set the duration until the next work event

Note: The only reason this code is place here is because the work activity and the commute activity use it.

Parameters **clock** (temporal.Temporal) – the current time

Returns the duration [minutes] until the next minutes

Return type int

get_current_meal(*time_of_day*)

This function gets the closest meal to the time of day.

Parameters **time_of_day** (*int*) – the time of day

Returns return the meal

Return type *meal.Meal*

get_meal(*id_meal*)

Get the specific meal given by a meal identifier.

Parameters **id_meal** (*int*) – the meal identifier

Returns the meal given by the id

Return type *meal.Meal*

get_next_meal(*clock*)

This function gets the next meal. The meal must occur after the current time.

Parameters **clock** (temporal.Temporal) – the current time

Return the_meal the next meal

Return type *meal.Meal*

print_child_status()

This function represents the child status as a string.

Return msg the child/ adult status

Return type str

set_child_flag(*age*)

Sets the flag indicating whether a person is a child.

Parameters **age** (*int*) – the age of the person [years]

Returns None

set_job(*job_id*, *dt=0*)

This function sets the job and the alarm time (if used) that corresponds to the job. The alarm is set, if a person is using the alarm.

Parameters

- **job_id** (*int*) – job identifier
- **dt** (*int*) – the amount of time before the job start.

Returns None

set_work_alarm(*dt=0*)

This sets the alarm time due to work

If a person uses an alarm, the alarm is set to be "dt" minutes before work time

Parameters **dt** (*int*) – the amount of time to wake up before the work event [minutes]

Returns None

test_func (*time_of_day*, *the_meal*)
This is used for testing.

Note: This function has no real purpose and will be deleted in future versions.

Parameters

- **time_of_day** (*int*) – the time of day in minutes
- **the_meal** (meal.Meal) – a meal object

Returns None

toString ()
Represents the Social object as a string.

Returns the representation of the Social object

Return type str

2.25 state module

This module contains code that governs information relevant to a Person's state.

This module contains class `state.State`.

class state.**State** (*status=0*)
Bases: object

This class contains information relevant to a Person state

Parameters **status** (*int*) – the status of the Person

Variables

- **'activity'** (activity.Activity) – the particular Activity of the Asset
- **arg_start** (*list*) – the list of arguments for the start() function
- **arg_end** (*list*) – the list of arguments for the end() function
- **'asset'** (asset.Asset) – the Asset that is being used
- **asset_list** (*list*) –
- **is_init** (*bool*) – this is a flag indicating whether or not the Agent is in the initialization state. This state only occurs during the first step of the simulation.
- **status** (*int*) – the status of a Person
- **t_end** (*int*) – the end time of a state [minutes, universal time]
- **t_start** (*int*) – the start time of the current state [minutes, universal time]
- **round_dt** (*int*) – the amount of minutes [-1, 0, 1] to round an activity duration
- **dt_frac** (*float*) – the fraction of a minutes subtracted from rounding down from the true projected activity duration
- **do_interruption** (*bool*) – a flag indicating whether the Person is interrupting an ongoing activity

end_activity()
This function ends an activity.

Returns None

halt_activity(*p*)
This function runs the halt activity. The function is used by interruptions to stop an activity **immediately** without giving benefits to the need that the halted activity addressed.

Parameters **p** (person.Person) – the person of interest

Returns None

print_activity()
The string representation of the activity. This function handles the possibility of the activity being None.

Returns the representation of the activity

Return type str

print_asset()
This function represents the asset as a string. This function handles the possibility of the asset being None.

Returns the representation of the asset

Return type str

print_status()
This function represents the status as a string.

Returns the representation of the status

Return type str

reset(*t_univ*)
Reset the state object to the default behavior at the beginning of the simulation.

Parameters **t_univ** (*int*) – the time of the beginning of the simulation in universal time [seconds]

Returns None

reset_rounding_parameters()
This function resets the rounding parameters to zero.

Returns None

reset_time_status(*t_start*, *status=0*)
This function resets the time information to the current time and sets the status. This function is usually used at the end of an activity.

Parameters

- **t_start** (*int*) – the start time [minutes, universal time]
- **status** (*int*) – the status of the person

Returns None

run_activity(*arg*, *func*)
This function allows an activity to start, end, or halt

Parameters

- **arg** (*list*) – arguments for the func() function
- **func** (*function*) – arguments for the func() function

Returns None

start_activity()
This function starts an activity

Returns None

toString()
This function represents the State object as a string.

Returns the representation of the State object

Return type str

2.26 temporal module

This file contains code that handles the time related aspects of this code.

This file contains code for class *temporal.Temporal*. This file also includes other functions that are accessed outside of the Temporal class.

Module author: Dr. Namdi Brandon

class temporal.**Temporal**(*t_univ=0*)
Bases: object

This class handles all the time keeping responsibilities.

Universal time is the total amount of time in minutes elapsed from the start of the calendar year.

Day 0 at 0:00 corresponds to a universal time of 0

Day 1 at 0:00 corresponds to a universal time of 1 * 24 * 60

Day 359 at 0:00 corresponds to a universal time of 359 * 24 * 60

Parameters **t_univ** (*int*) – the time in universal time [minutes]

Variables

- **day** (*int*) – the day number in the simulation
- **day_of_week** (*int*) – a number 0, 1, 2, ... 6 corresponding to days of the week where 0 is Sunday, 1 is Monday, ... 6 is Saturday
- **dt** (*int*) – the step size in the simulation [minutes] (**antiquated**)
- **hour_of_day** (*int*) – the hour of the day [0, 23]
- **is_weekday** (*bool*) – a flag indicating if it's a weekday (Monday-Friday) if True. False, otherwise.
- **is_night** (*bool*) – a flag indicating if the time of day is after DUSK and before DAWN if True. False, otherwise.
- **min_of_day** (*int*) – the minute of the day [0, 60 - 1]
- **t_univ** (*int*) – the universal time [minutes]
- **time_of_day** (*int*) – the time of the day [minutes], [0, 1, ... 24 * 60 -1]
- **season** (*int*) – the season
- **tic** (*int*) – indicates that current tick (each tick corresponds to a step of size dt)
- **step** (*int*) – indicates the current step in the simulation [0, ... num_steps-1]

print_day_night()
Represents whether it's day or night as a string

Return msg daytime / nighttime status (or an error message, if there is an error)

Return type str

print_day_of_week()
Represents the day of the week as a string

Return msg the day of the week (or an error message, if there is an error)

Return type str

print_season()
Represents the seasons as a string

Returns the season (or an error message, if there is an error)

Return type str

print_time_of_day_to_military()
Represents the time of day as military time.

Returns the time of day in military time

Return type str

reset(*t_univ*)
Reset the temporal object to the initial state.

Parameters **t_univ** (*int*) – The time [seconds, universal time] that the time should be reset to

Returns

set_day_of_week()
This function sets the day of the week.

In addition, this function sets the day count, the day of the week, and a flag indicating whether it is a weekday or not.

Returns None

set_season()
This function sets the season.

Day 0 is the beginning of winter

Returns None

set_time()
This function sets all the time variable due to the universal time.

This function sets

1. the time of day
2. the day of the week
3. the season
4. the tic.

Returns None

set_time_of_day()

Given the universal time, this function sets the time of day in minutes.

Returns None

toString()

This function represents the Temporal object as a string.

Return msg the representation of the temporal object

Return type str

update_time()

Increments the time by 1 time step.

> **Warning:** This function is outdated!

Returns None

temporal.**convert_cyclical_to_decimal**(*t*)

This function converts cyclical time to decimal time

Parameters **t** (*int*) – the time of day [minutes]

Return out the time of day in [hours]

Return type float

temporal.**convert_cylical_to_universal**(*day*, *time_of_day*)

This function converts a cyclical time to the universal time.

Parameters

- **day** (*int*) – the day of the year
- **time_of_day** (*int*) – the time of day [minutes]

Return t the time in universal time

Return type int

temporal.**convert_decimal_to_min**(*t*)

This function takes in the time of day as a decimal and outputs the time in minutes

Parameters **t** (*float*) – the time of day [0, 24) [hours]

Return out the time of day [minutes]

Return type int

temporal.**convert_universal_to_decimal**(*t_univ*)

This function takes in the universal time and converts it to the time of day in decimal format [0, 24)

Parameters **t_univ** (*int*) – the universal time [minutes]

Return out the universal time [hours]

Return type float

temporal.**print_military_time**(*t*)

Represents the time of day in military time assume that time is in minutes format.

Parameters **t** (*int*) – the time of day [minutes]

Return msg the time of day in military time 00:00

Return type str

2.27 transport module

This module contains information about the `asset.Asset` that allows a `person.Person` to do the following:

1. `commute.Commute_To_Work`
2. `commute.Commute_From_Work`

activities. This module contains code for `transport.Transport`.

Module author: Dr. Namdi Brandon

class `transport.`**`Transport`**

Bases: `asset.Asset`

This class is an asset that allows for commuting.

Activities in this asset:

1. `commute.Commute_To_Work`
2. `commute.Commute_From_Work`

`initialize`(*people*)

This function sets the transport location according to whether or not the Person is commuting to or from work.

Note: This function just sets the transport object to be at the home

Parameters **`people`** (*list*) – a list of people in the simulation

Returns None

2.28 travel module

This module contains code for the `need.Need` associated with the desire to move from one environment to another.

This file contains code for `travel.Travel`.

Module author: Dr. Namdi Brandon

class `travel.`**`Travel`**(*clock*, *num_sample_points*)

Bases: `need.Need`

This class governs the need for traveling.

Parameters

- **`clock`** (temporal.Temporal) – the time
- **`num_sample_points`** (*int*) – the number of temporal nodes in the simulation

`decay`(*p*)

This function decays the satiation. Travel for commuting only decays when the work need is low

Parameters **`p`** (person.Person) – the person whose satiation is decaying

Returns None

`decay_work_commute`(*p*)

This decays the satiation level in order to commute to work. For the satiation to decay the person needs the following

1.The agent should leave the home to go to work

2.The agent should leave work to go home

Parameters **p** (person.Person) – the person of interest

Returns None

initialize(*p*)

This function initializes the Travel by updating the *scheduler.Scheduler* for Travel

Parameters **p** (person.Person) – the person of interest

Returns None

perceive(*clock*, *job*)

This function gives the satiation for Travel if the Travel need is addressed now.

Note going to work can only happen according to work hours of the job.

Parameters

- **clock** (temporal.Temporal) – the time the need to travel is perceived
- **job** (occupation.Occupation) – the job of the person

Return mag the perceived magnitude of the need

Return type float

2.29 universe module

This module contains code that is responsible for running the simulation. This file contains *universe.Universe*. The Universe contains all agents and objects. The Universe is responsible for running the simulation itself.

Module author: Dr. Namdi Brandon

class universe.**Universe**(*num_steps*, *dt*, *t_start*, *num_people*)

Bases: object

The Universe is the governing engine of the simulation.

Parameters

- **num_steps** (*int*) – the number of time steps in the simulation
- **dt** (*int*) – the step size in the simulation [minutes]
- **t_start** (*int*) – the start time for the simulation [minutes, universal time]
- **num_people** (*int*) – the number of people in the household

Variables

- **clock** (temporal.Temporal) – does the timekeeping in the simulation
- **"home"** (home.Home) – the home the Persons live in
- **people** (*list*) – a list of all Person objects created in the Universe object
- **t_start** (*int*) – the start time for the simulation [minutes, universal time]
- **t_end** (*int*) – the last time for the simulation [minutes, universal time]
- **schedule** (scheduler.Scheduler) – the schedule governing each agent's needs

address_needs(*do_interruption=False*)

This function checks the needs of the agents

The function uses a recursion loop to choose activities.

The Recursion:

1. Gather all of the advertisements (object-person pairings)
2. Assigns 1 activity to the Person with the highest score.
3. That Person starts the activity, thereby updating the state of available activities in the home.
4. The recursion starts again, where the Home advertises to all remaining Person(s).

Note If no activity will be done this time step to a Person, a Person is set to the temporary status `state.IDLE_TEMP`, so that the Home knows not to advertise to that Person.

Parameters **do_interruption** (*bool*) – this flag indicates whether or not advertisements should be made for activities that will interrupt the current activity (if True). If False, the advertisements are made for non-interrupting activities.

Returns None

advertise(*do_interruption=False*)

This function obtains a list of all of the possible activities each person could potentially start in this time step.

Parameters **do_interruption** (*bool*) – this flag indicates whether to make advertisements due to an interrupting activity (if True) or not (if False).

Return ads ads is a list of dictionaries for advertisements:

Dictionary (score, asset, activity, person) containing the various data for each advertisement: (score, asset, activity, person) coupling

Return type list

check_expired_activities()

This function checks for expired activities. If found, end the activities.

Returns None

decay_needs(*dt=None*)

This function decays the needs according to the default behavior. That is, assume the needs are not addressed earlier.

Parameters **dt** (*int*) – the number of minutes to decay the needs by. The default behavior is to use the scheduler's time. If a number is specified, then it should be the number of minutes until the end of the simulation.

Returns None

initial_step()

This function is supposed to run the first time step of the run() loop

1. store the current time
2. address the needs assuming interruption
3. address the needs assuming NO interruption
4. update the history
5. update the clock

6.decay the needs

Note: this function is **NOT** called on in the current implementation yet

Returns None

initialize_needs()

This function initializes the need state of each Person at the beginning of simulation based on the current time.

The needs are initialized in this order (the order matters)

1.Rest

2.Hunger

3.Income

4.Travel

5.Interruption

Returns None

print_activity_info(*p*)

This function stores activity info used for testing / developing/ debugging as a string.

Parameters **p** (person.Person) – the person of interest

Returns None

reset(*t_univ*)

This code resets the simulation by initializing the agents, home, and clock to the beginning status of the simulation.

This code does the following:

1.reset the clock

2.reset the home

3.reset each person

4.initialize each person

5.initialize the home

Parameters

- **p** (params.Params) – the parameters
- **t_univ** (*int*) – the time of the beginning of the simulation [seconds]

Returns

run()

This function is responsible for running the simulation. Instead of running the simulation minute-by-minute, in an effort to reduce run-time, the simulation skips time steps and addresses the agent at times that actions should occur. These times are dictated by the scheduler.

The function proceeds as following:

While the current time is less than the final time

1.check for expired activities for all agents. If activities should have expired, tell the agent to end them

2.start new activities by addressing the needs for all agents (assuming no interruption)

3.decay the satiation for Interruption for all agents

4.start new activities by addressing the needs for all agents (assuming interruptions only)

5.update the history of the status of each agent

6.find the next time to jump to in the simulation according to the scheduler

7.update the clock to the new time

8.decay the needs for all agents

9.Repeat

For the last time step

1.update the clock

2.decay the needs for each agent

3.update the history of the status of each agent

Note: I must change N_MAX to N_MAX = DAY_2_MIN * 365

Returns

`select_activity`(*ads*)

Given a list of activity advertisements, this function selects the Person with the largest activity score and outputs the score, asset, activity, and person.

Parameters **`ads`** (*list*) – a list of advertisements for this time step

Return chosen the selected activity advertisement (score, asset, activity, person)

Return type dict

`set_alarm`()

This function sets the alarm for those Person(s) who use an alarm

---

Note: This function is **NOT** used. There is currently no alarm capability.

Returns None

`test_func`()

Note: This function is just for debugging.

Returns

`toString`()

Represent the Universe object as a string.

This function outputs the representation of:

1.the clock

2. the home
3. agent person residing in the home

Return msg a representation of the Universe object

Return type str

update_clock (*t*)

This function updates the clock by

1. setting the clock to the given time
2. updating the step of the simulation
3. storing the history of the time nodes used in the simulation

Parameters **t** (*int*) – the time the clock should be set to

Returns

update_history (*step*)

Update the histories for each Person by storing the following:

1. the current state's status
2. the current activity
3. the current satiation value for each needs
4. the current location

Parameters **step** (*int*) – the time step

Returns None

update_history_new ()

Update the histories of each person.

Returns None

2.30 work module

This module contains code that governs the *activity.Activity* that gives a Person the ability to go to work/ school.

This file contains *work.Work*.

Module author: Dr. Namdi Brandon

class work.**Work**

Bases: *activity.Activity*

This class allows a Person to work / go to school in order to satisfy the need *income.Income*.

advertise (*p*)

This function calculates the score of the advertised work activity to a Person

Parameters **p** (person.Person) – the person of interest

Return score

Return type float

end(*p*)
This function handles the end of an Activity

Parameters **p** (person.Person) – the person of interest

Returns None

end_work(*p*)
This function sets the variables pertaining to coming back from work by doing the following:

1. free the asset from use
2. set the asset's state to `state.IDLE`
3. set the Income satiation to 1
4. decay the need Travel
5. sample the new work start time
6. sample the new work end time
7. update the scheduler to take into account the next work event

Parameters **p** (person.Person) – the person of interest

Returns None

halt(*p*)
This function handles an interruption of an Activity.

Parameters **p** (person.Person) – the person of interest

Returns None

halt_work(*p*)
This function interrupts the work behavior by doing the following:

1. frees the current asset
2. the asset's state is set to `state.IDLE`
3. the Interruption satiation is set to 1.0
4. the Interruption's activity start/ stop

Note No benefits of working are given while being interrupted

Parameters **p** (person.Person) – the person of interest

Returns None

set_end_time(*p*)
Calculates the end time of work.

Parameters **p** (person.Person) – the person of interest

Return t_end the end time [minutes, universal time]

Return type int

start(*p*)
This handles the start of an Activity

Parameters **p** (person.Person) – the person of interest

Returns None

start_work(*p*)

This function starts the work activity

- updates that asset's status and number of users
- changes the location of the Person
- updates that person's status
- calculates the end time of the work activity
- update the scheduler for the Income satiation
- update the scheduler for the Travel satiation
- set the day for the work period

Parameters **p** (person.Person) – the person of interest

Returns None

test_func(*p*)

Note: This function is **NOT** used.

Parameters **p** (person.Person) – the person of interest

Returns

2.31 workplace module

This module contains code for the *asset.Asset* that allows a Person to go to work / school.

This file contains *workplace.Workplace*.

Module author: Dr. Namdi Brandon

class workplace.**Workplace**

Bases: *asset.Asset*

This class allows a Person to go to work / school.

Activities in this asset: *work.Work*

CHAPTER 3

Run Directory

These are the files needed to run an instance of ABMHAP with one agent parametrized by user-defined parameters.

The driver for these type of runs is main.py.

Contents:

3.1 main module

This is code is runs the simulation for the Agent-Based Model of Human Activity Patterns (ABMHAP) module of the Life Cycle Human Exposure Model (LC-HEM) project.

In order to run the code, do the following:

1. set the user-defined parameters of the simulation in main_params.py
2. **run the code via** > python main.py

Note: In order to run the debugger do the following in windows:

> python -m pdb main.py

Module author: Dr. Namdi Brandon

main.**get_diary**(*u*)

This function output the result of the simulation in terms of an activity diary.

Parameters **u** (universe.Universe) – the governing engine of the simulation

Returns the activity diary describing the behavior of the agent

Return type *diary.Diary*

main.**plot_cdfs**(*d*, *keys*)

This function plots the cumulative distribution function of start time, end time, and duration for each activity in the the simulation.

Parameters

- **d** (diary.Diary) – the results of the simulation
- **keys** (*list*) – list of activities to graph

Returns

main.**plot_histograms**(*d*, *keys*)

This function plots the histograms of start time, end time, and duration for each activity in the the simulation.

Parameters

- **d** (diary.Diary) – the results of the simulation
- **keys** (*list*) – list of activities to graph

Returns

main.**save_output**(*df*, *fname*)

This function saves the output of the simulation.

Parameters

- **df** (*pandas.core.frame.DataFrame*) – the activity-diary output of the simulation
- **fname** (*str*) – the file name of the saved file. It must end with ".csv"

3.2 main_params module

This module is responsible for containing parameters that main.py uses to control the simulation.

The user should set the parameters in this module **before** running the driver (main.py)

Module author: Dr. Namdi Brandon

main_params.**set_no_variation**(*num_people*)

This function sets the standard deviations in all of the activity-parameters to zero.

Parameters **num_people** (*int*) – the number of people in the simulation

Returns a tuple of the standard deviations of all of the activity-parameters

3.3 scenario module

This file contains information to run the Agent-Based Model of Human Activity Patterns (ABMHAP) in in different simulation scenarios in which the agent has a user-defined parametrization.

The following classes are in this module

1. *scenario.Scenario*
2. *scenario.Solo*
3. *scenario.Duo*

Module author: Dr. Namdi Brandon

class scenario.**Duo**(*hhld_params*)

Bases: *scenario.Scenario*

This class parametrizes / runs a simulation scenario for the cases where two Singleton (*singleton.Singleton*) persons live in the same residence.

Note: This scenario is used in order to check for activity conflicts among 2 agents living in the same household. Currently it is used primarily as a debugging tool.

Parameters **hhld_params** (params.Params) – the parameters for the household that contain relevant information for the simulation

class scenario.**Scenario**(*hhld_params*)

Bases: object

This class governs what a simulation scenario consists of.

Parameters **hhld_params** (params.Params) – the parameters for the household that contain relevant information for the simulation

Variables

- **id** (*int*) – the scenario identifier number
- **u** (universe.Universe) – the universe object for the simulation
- **'params'** (params.Params) – the parameters needed that control the simulation

activity_diary()

This function returns the activity diary for each person

Each person will attain the following tuple

1. grouping of the index for each activity
2. the day, (start-time, end-time), activity code, and location for each activity-event, in a numeric format
3. the same as above in a string format

Returns

default_location()

Sets the default location for all Person's to be be at the home.

This location may be overridden later in the initialization of Persons.

Returns None

initialize()

This function initializes the scenario before the simulation scenario is run

More specifically, the function does the following:

1. Sets the state and location for each person
2. Sets the home
3. Initialize the initial need-association states for the Person(s) and Home

Returns None

run()

This function initializes the scenario and then runs the ABMHAP simulation.

Returns None

set_home()

This function sets aspects of the home in order to run the simulation scenario.

More specifically, the function does the following

1. set the home revenue
2. set the home population

Returns None

set_state()

This function initializes the scenario in order to run the simulation.

More specifically, this function does the following:

1. **For each Person, the following is set:**
   (a) identification number
   (b) the state

Returns None

class scenario.**Solo**(*hhld_params*)

Bases: *scenario.Scenario*

This class parametrizes / runs a simulation scenario for the Singleton (*singleton.Singleton*) person.

Parameters **hhld_params** (params.Params) – the parameters for the household that contain relevant information for the simulation

3.4 singleton module

This file contains information for creating the default agent that represents a person that lives alone in the home. Singleton will be the name of this type of agent.

This module contains *singleton.Singleton*.

Module author: Dr. Namdi Brandon

class singleton.**Singleton**(*house*, *clock*, *schedule*)

Bases: *person.Person*

Singleton default is a person that has the following characteristics

1. female
2. 30 years old
3. goes to bed at 22:00 and sleeps for 8 hours
4. lives alone and has no children
5. works the Standard Job
6. eats breakfast at 7:30 for 15 minutes, lunch at 12:00 for 30 minutes, and dinner at 19:00 for 45 minutes

Parameters

- **house** (home.Home) – the place of residence
- **clock** (temporal.Temporal) – the clock running in the simulation
- **schedule** (scheduler.Scheduler) – the schedule for the agent

print_params()

This function prints the activity-parameter means in chronological order of start time. This results in the ability to print the mean daily routine.

Returns a representation of the parameters of the agent in increasing values of start time

Return type str

set (*param*, *idx*)

This function sets the Singleton's parameters.

The function does the following

1. Sets the biology
2. Sets the job information
3. Sets the alarm
4. Sets the meal information

Parameters

- **param** (params.Params) – parameters describing the household
- **idx** (*int*) – the respective index number of the Person of interest in the household

Returns None

CHAPTER 4

Indices and tables

- genindex
- modindex
- search

a
activity, 9
asset, 11

b
bed, 12
bio, 12

c
commute, 14

d
diary, 18

e
eat, 21

f
food, 25

h
home, 26
hunger, 27

i
income, 30
interrupt, 31
interruption, 32

l
location, 33

m
main, 75
main_params, 76
meal, 34
my_globals, 36

n
need, 39

o
occupation, 40

p
params, 46
person, 51

r
rest, 53

s
scenario, 76
scheduler, 56
singleton, 78
sleep, 57
social, 59
state, 62

t
temporal, 64
transport, 67
travel, 67

u
universe, 68

w
work, 72
workplace, 74
